# Rapid SDS/trypsin decellularization of rat submandibular gland yields an ECM scaffold supporting salivary gland tissue engineering

**DOI:** 10.3389/fbioe.2026.1795289

**Published:** 2026-04-28

**Authors:** Jie Gao, Bo Kyoung Kang, Xiuxia Wang, Minyan He, Zhaoqi Yuan, Shun Yu, Lin Lu, Jun Yang

**Affiliations:** 1 Department of Plastic and Reconstructive Surgery, Shanghai Ninth People’s Hospital, Shanghai Jiao Tong University School of Medicine, Shanghai, China; 2 Shanghai Institute for Plastic and Reconstructive Surgery, Shanghai Ninth People’s Hospital, Shanghai Jiao Tong University School of Medicine, Shanghai, China; 3 Department of Burns and Plastic Surgery, The Affiliated Hospital of Jiangnan University, Wuxi, China

**Keywords:** decellularized extracellular matrix, extracellular matrix scaffold, rapid decellularization, salivary gland tissue engineering, stem cell recellularization, submandibular gland, tissue engineering

## Abstract

**Introduction:**

Salivary gland hypofunction caused by radiation, Sjögren’s syndrome, or other insults leads to xerostomia and currently lacks effective regenerative treatments. Decellularized organ-specific extracellular matrix (ECM) scaffolds offer a promising strategy toward gland restoration by recapitulating the native microenvironment.

**Methods:**

In this study, we developed a rapid decellularization protocol for rat submandibular glands (SMGs) to create a biomimetic ECM scaffold for salivary gland tissue engineering. Following freeze–thaw pretreatment, detergent and enzyme incubations totaled 4 h and the overall detergent/enzyme processing time, including intermediate PBS washes, was approximately 5 h. The resulting scaffold was characterized by histology, immunostaining, biochemical analysis, and proteomic analysis. Recellularization of the decellularized SMG (dSMG) was performed via intraductal injection of human submandibular gland mesenchymal stem cells (hMSCs) or human submandibular gland stem cells (hSMG-SCs), followed by *in vitro* culture. In addition, hSMG-SC-seeded dSMG scaffolds were implanted subcutaneously in immunodeficient mice for *in vivo* evaluation.

**Results:**

The protocol achieved effective decellularization, reducing residual DNA to <50 ng/mg dry tissue while preserving essential matrix components. The resulting dSMG scaffold retained key structural proteins, including collagens I and IV, laminin, and fibronectin, as well as glycosaminoglycans, as confirmed by histology, immunostaining and proteomic analysis. Recellularization of the dSMG resulted in cell repopulation, viability and proliferation over 7 days in culture. The hMSCs remained viable and upregulated genes associated with matrix remodeling, whereas hSMG-SCs maintained expression of epithelial markers, e.g., Cytokeratin 7, within the scaffold microenvironment. When hSMG-SC-seeded dSMG scaffolds were implanted subcutaneously into immunodeficient mice for 8 weeks, they became well-vascularized and supported CK7-positive duct-like epithelial organization with persistence of human cell signal, together with weak focal AQP5 expression, whereas these features were not observed in acellular controls.

**Discussion:**

These findings demonstrate that the dSMG scaffold can provide a favorable niche for cell survival and early glandular tissue organization, highlighting its potential as a biomaterial platform for salivary gland tissue engineering.

## 1 Introduction

Salivary glands are exocrine organs that secrete saliva containing antimicrobial factors, buffering systems, lubricants, and enzymes that are essential for oral homeostasis and digestion (Lynge Pedersen and Belstrøm, 2019; Matsuoka et al., 2025; Yoon et al., 2022). These glands play a pivotal role in maintaining oral health, facilitating mastication, and protecting mucosal surfaces. Multiple pathological conditions—including head-and-neck radiotherapy, Sjögren’s syndrome, oral tumor resection, systemic diseases, and the use of certain medications—can cause salivary gland hypofunction, leading to reduced salivary output and xerostomia, which significantly impacts patients’ quality of life(Rose et al., 2023; Song et al., 2024).

Current clinical therapies primarily provide symptomatic relief through saliva substitutes, lubricants, and sialogogues, rather than true functional restoration of glandular tissue (Li et al., 2022; Nascimento et al., 2019). Consequently, there is an urgent need for regenerative strategies capable of reconstructing functional salivary gland tissue to achieve lasting restoration of secretion capacity (Lilliu et al., 2016; Rose et al., 2023).

A central challenge in tissue engineering lies in developing a supportive microenvironment that accurately recapitulates the extracellular matrix (ECM), where cells can adhere, proliferate, and differentiate (Brown et al., 2022). The native ECM provides organ-specific biochemical and biophysical cues that regulate cell behavior and maintain tissue integrity. Composed of structural proteins such as collagens, laminins, proteoglycans, and fibronectin, the ECM not only preserves tissue architecture but also stores and presents growth factors that guide cell fate decisions (Gao et al., 2014).

Decellularized extracellular matrix (dECM) scaffolds have thus gained considerable attention for tissue regeneration, as they preserve native ECM composition and ultrastructure while removing immunogenic cellular components (Guo et al., 2024). dECM-based biomaterials derived from skin, nerve, intestine, and multiple solid organs—including heart, lung, liver, kidney, and mammary gland—have demonstrated excellent biocompatibility and translational potential in tissue engineering and regenerative medicine (Brown et al., 2022; Golebiowska et al., 2024). However, studies specifically focusing on salivary gland dECM remain limited.

Existing protocols for submandibular gland (SMG) decellularization are often complex or time-intensive, with typical detergent exposure lasting 24–32 hours—for instance, Gao et al. used 10% SDS for 32 h (Gao et al., 2014), and Albusaily et al. applied a Triton X-100/SDS cocktail for 24 h (Albusaily et al., 2025). Importantly, in whole-organ decellularization, effective cell removal must be balanced against preservation of the biochemical and ultrastructural features of the native matrix. Prolonged exposure to ionic detergents such as SDS has been reported to alter collagen fiber organization, reduce glycosaminoglycan retention, and damage basement membrane-associated components, thereby compromising the instructive microenvironment needed for subsequent recellularization (Crapo et al., 2011; Faulk et al., 2014; Poornejad et al., 2016; He et al., 2017). Because basement membrane proteins such as laminin and collagen IV are critical for epithelial attachment, polarity, and tissue-specific cell behavior, excessive detergent treatment may be particularly detrimental in glandular organs that depend on preserved epithelial–matrix interactions (Faulk et al., 2014; Lopera Higuita et al., 2022).

From a translational perspective, lengthy detergent-based protocols may also hinder process standardization, reduce manufacturing efficiency, and increase batch-to-batch variability, all of which are relevant barriers for scalable production of clinically applicable dECM scaffolds (Golebiowska et al., 2024; Jin et al., 2025; Yang et al., 2025). Therefore, a rapid decellularization strategy is not only desirable for shortening preparation time, but also for minimizing detergent-associated ECM damage while improving practicality and reproducibility for future bioengineering applications.

In this study, we developed a rapid and practical decellularization protocol for rat submandibular glands (SMGs) that shortens detergent and enzymatic exposure time while preserving ECM integrity. The resulting decellularized SMG (dSMG) scaffolds were comprehensively characterized for matrix composition and ultrastructure. Their recellularization potential was evaluated by intraductal seeding of human submandibular gland mesenchymal stem cells (hMSCs) or submandibular gland stem cells (hSMG-SCs) under dynamic culture. Furthermore, dSMG scaffolds pre-seeded with hSMG-SCs were implanted subcutaneously in nude mice to assess *in vivo* integration. Collectively, our results demonstrate that the optimized dSMG scaffold preserves key ECM components, supports cell repopulation, and promotes vascular ingrowth with duct-like epithelial organization *in vivo*, underscoring its promise as a bioactive scaffold for salivary gland tissue engineering.

## 2 Materials and methods

### 2.1 Ethics and animals

All animal procedures were performed in accordance with the NIH Guide for the Care and Use of Laboratory Animals and are reported in line with the ARRIVE guidelines. The study was approved by the Ethics Committee of the Ninth People’s Hospital of Shanghai Jiao Tong University School of Medicine (approval No. SH9H-2023-A823-1). Male Sprague–Dawley rats (6–8 weeks) and male BALB/c nude mice (4–6 weeks) were purchased from Shanghai Bicaikewing Co., Ltd. Animals were maintained under SPF conditions with controlled temperature and humidity. Euthanasia was performed by carbon dioxide inhalation using an laboratory animal euthanasia device equipped with CO_2_ concentration and flow monitoring(Shanghai MinlyLab Hi-Tech Development Co., Ltd, China). Species-specific settings were used as follows: for mice, the chamber was set to 30% CO_2_ with a flow rate of 8 L/min and a holding time of 120 s; for rats, the chamber was set to 40% CO_2_ with a flow rate of 10 L/min and a holding time of 300 s. After CO_2_ exposure, cervical dislocation was performed as the secondary physical method to confirm death, in accordance with institutional guidelines.

### 2.2 Decellularization of SMG

Submandibular glands were harvested from 6–8-week-old male Sprague–Dawley rats immediately after euthanasia and briefly rinsed in cold phosphate-buffered saline (PBS; GIBCO, USA). Tissues underwent three freeze–thaw cycles, rapid freezing in liquid nitrogen followed by thawing at 37 °C. All subsequent steps were performed on an orbital shaker at 200 rpm. All subsequent procedures were performed under aseptic conditions. Each gland was processed in a 15 mL centrifuge tube containing 15 mL of reagent or PBS. Samples were immersed in 1% (w/v) sodium dodecyl sulfate (SDS; Sigma-Aldrich, USA) for 3 h, followed by three sequential washes in 15 mL PBS, 10 min each, with buffer replacement every 10 min, and then treated with 0.25% trypsin–EDTA (GIBCO, USA) at 37 °C for 0 h, 1 h or 2 h, followed by a second series of three sequential washes in 15 mL PBS under the same conditions. Based on trypsin exposure time, three protocols were defined as S3, S3T1 and S3T2.

In preliminary screening, additional conditions (S1T2, S2T1 and T3) were evaluated by H&E staining, and conditions showing incomplete decellularization were not used for downstream experiments. Decellularized scaffolds were sterilized by immersion in an antibiotic–antimycotic solution containing penicillin (10,000 U/mL), streptomycin (10,000 μg/mL) and amphotericin B (25 μg/mL) for 12 h under agitation, washed thoroughly with PBS, and stored at 4 °C until use.

### 2.3 Histology and immunostaining

Tissues were fixed in 4% paraformaldehyde at 4 °C for 24 h, dehydrated in graded ethanol, embedded in paraffin and sectioned at 5 μm. For histological evaluation, deparaffinized sections were stained with hematoxylin and eosin (H&E; Servicebio, China). Masson’s trichrome staining was performed using a commercial kit (Solarbio, China) according to the manufacturer’s instructions.

For collagen I immunohistochemistry, sections were deparaffinized and rehydrated, blocked with 5% bovine serum albumin (BSA) for 30 min, and incubated overnight at 4 °C with an anti-collagen I primary antibody (Abcam, USA). Sections were then incubated with an HRP-conjugated secondary antibody (Proteintech, USA), and signal was developed using a DAB detection kit (Gene Tech, China).

For immunofluorescence staining, sections were deparaffinized, rehydrated and blocked with 1% BSA for 30 min, followed by overnight incubation at 4 °C with primary antibodies against collagen I (Abcam, USA), collagen IV, laminin or fibronectin (Proteintech, USA). After washing with PBS, sections were incubated with Alexa Fluor 488–conjugated secondary antibodies (EMAR, China) for 1 h at 37 °C in the dark. Nuclei were counterstained with DAPI for 5 min prior to imaging.

### 2.4 Biochemical analysis of native SMG and decellularized scaffolds

Native SMG and decellularized scaffolds (S3, S3T1 and S3T2) were lyophilized and weighed before analysis.

Genomic DNA was extracted from 10 to 25 mg of lyophilized tissue using a commercial genomic DNA purification kit (TIANGEN, China) following the manufacturer’s instructions. DNA concentration was quantified using the Quant-iT PicoGreen dsDNA assay kit (Invitrogen, USA), and DNA content was normalized to dry tissue weight (ng DNA/mg dry tissue).

Total collagen content was determined using a hydroxyproline assay kit (Nanjing Jiancheng Bioengineering Institute, China). Briefly, freeze-dried samples were hydrolyzed and processed according to the manufacturer’s protocol, and the absorbance of the reaction product was measured at 550 nm using a microplate reader. Collagen content was calculated from a hydroxyproline standard curve and expressed as µg collagen per mg dry tissue.

Sulfated glycosaminoglycan (GAG) content was measured using a dimethylmethylene blue (DMMB, Daixuan Biology, China)-based colorimetric method. Lyophilized samples were homogenized and processed according to the kit protocol, and 1,9-dimethylmethylene blue dye was added to form GAG–dye complexes. Absorbance was recorded at 525 nm using a microplate reader. GAG concentrations were calculated from a chondroitin sulfate standard curve and normalized to sample dry weight.

### 2.5 Reverse transcription–polymerase chain reaction (RT-PCR) and agarose gel electrophoresis

Total RNA was extracted from native SMG and dSMG tissues using TRIzol reagent (Sigma-Aldrich, USA) following the manufacturer’s instructions. RNA concentration and purity were measured using a NanoDrop spectrophotometer (Thermo Fisher Scientific, USA). One microgram of RNA was reverse-transcribed into cDNA using the High-Capacity cDNA Reverse Transcription Kit (Thermo Fisher Scientific, USA) in a 20 μL reaction system. Primer sequences used in this study are provided in Supplementary Table S1.

PCR amplification was performed using 2× PCR premix (e.g., Taq Master Mix, Vazyme, China) in a 25 μL reaction containing 12.5 μL premix, 1 μL forward primer, 1 μL reverse primer, 2 μL cDNA template, and nuclease-free water. The PCR cycling conditions were: initial denaturation at 95 °C for 3 min, followed by 30 cycles of 95 °C for 30 s, 58 °C–60 °C for 30 s (optimized for each gene), and 72 °C for 30 s, with a final extension at 72 °C for 5 min.

PCR products were separated by electrophoresis on a 2% agarose gel containing ethidium bromide and visualized under UV illumination using a gel imaging system (Bio-Rad, USA). Band intensities were analyzed using ImageJ software.

### 2.6 Western blot

Total proteins were extracted from native SMG and dSMG samples using Total Protein Extraction kit (KeyGen Biotech, China). Tissues were homogenized and centrifuged at 12,000 rpm for 15 min at 4 °C. The supernatant was collected, and protein concentration was determined using a BCA Protein Assay Kit (Beyotime, China).

20 μg protein were mixed with loading buffer, denatured at 95 °C for 5 min, and separated by SDS–polyacrylamide gel electrophoresis (SDS-PAGE, CWBIO, China). Proteins were transferred onto polyvinylidene fluoride membranes (PVDF, Millipore, USA) using a wet-transfer system. Membranes were blocked with 5% skim milk (Corning, USA) for 1 h and then incubated overnight at 4 °C with primary antibodies against collagen I (Abcam, USA), collagen IV (Proteintech, USA), laminin (Abcam, USA), fibronectin (Proteintech, USA) and β-actin (Proteintech, USA). After TBST washing, membranes were incubated with HRP-conjugated goat anti-rabbit and goat anti-mouse secondary antibodies for 1 h at room temperature. Protein bands were visualized using enhanced chemiluminescence (ECL) substrate (Tanon, China) and imaged with a chemiluminescence detection system (Tanon-4600).

### 2.7 Proteomic analysis

Liquid chromatography-tandem mass spectrometry (LC-MS/MS) was performed using a data-independent acquisition (DIA) workflow to identify peptide sequences of native SMG and dSMG (n = 3), and subsequent proteomic analysis was used to characterize protein composition. DIA runs were processed using DIA-NN software, which applies neural networks and interference correction to achieve deep proteome coverage with stringent FDR control (Demichev et al., 2020).

To evaluate batch variation of extracellular matrix compositions, three batches from different donors were selected for each group. Lyophilized samples were trypsin digested and injected into LC-MS/MS, and the resulting DIA-MS/MS spectra were searched against the *Rattus norvegicus* reference proteome with a 1% FDR threshold.

Gene Ontology (GO) term and Kyoto Encyclopedia of Genes and Genomes (KEGG) pathway enrichment analyses were performed using DAVID Bioinformatics Resources, a comprehensive functional annotation tool for large gene/protein lists.

Matrisome proteins in native SMG and dSMG were classified based on the MatrisomeDB ECM-protein knowledge database, which provides curated ECM protein categories for proteomic annotation (Naba, 2023; Shao et al., 2023).

### 2.8 Human submandibular gland mesenchymal stem cells (hMSCs) and human submandibular gland stem cells (hSMG-SCs)

Human submandibular gland SMG tissues were obtained as surgical remnants from patients undergoing oral and maxillofacial surgery at the Ninth People’s Hospital of Shanghai Jiao Tong University School of Medicine. Sample collection was approved by the institutional ethics committee, and written informed consent was obtained from all participants. The Ethics Committee of the Ninth People’s Hospital of Shanghai Jiao Tong University School of Medicine approved the use of human samples. All procedures complied with the Declaration of Helsinki, and all participants provided written informed consent.

Fresh SMG specimens were transferred to a sterile culture dish in a biosafety cabinet and washed three times with phosphate-buffered saline to remove blood and adherent connective tissue. The tissue was trimmed and cut into glandular lobules, and the lobules were kept moist with 1 mL serum-free basal medium. The lobules were minced into approximately 0.3 mm^3^ explants using sterile ophthalmic scissors.

The explants were gently placed on the bottom of T25 flasks with an interval of about 5 mm. A small volume of complete medium was added to cover the flask bottom without dislodging the explants. The flasks were kept upright and incubated at 37 °C with 5% CO_2_ for 4–5 h to allow attachment. The flasks were then laid flat, and additional medium was added to fully immerse the explants. After 3 days, half of the medium was replaced to remove non-adherent fragments, and the medium was then changed every 3 days until colonies formed around the explants.

For hMSCs, cells were expanded and passaged in Mesenchymal Stem Cell Medium (MSCM, ScienCell, USA), when adherent cells reached about 85%–90% confluence. Cells were reseeded at a 1:3 split ratio and maintained at 37 °C with 5% CO_2_. Cells at passages 3–5 were used for subsequent experiments.

For hSMG-SCs, the isolation procedure was identical to that described above, but the culture medium was Keratinocyte Medium (KM, ScienCell, USA). Cells were maintained under the same incubation conditions and passaged when they reached approximately 85%–90% confluence. Cells at passages 3–5 were used for subsequent experiments.

### 2.9 Recellularization of dSMG

Before seeding, dSMG scaffolds were equilibrated in culture medium for 30 min hMSCs or hSMG-SCs (passages 3–5) were resuspended at 1 × 10^7^ cells/mL and injected into the main duct of each scaffold. Constructs were cultured in the corresponding medium at 37 °C with 5% CO_2_, with medium changes every 2 days, on an orbital shaker at 50 rpm.

### 2.10 Histological evaluation and Ki67 immunofluorescence of recellularized dSMG scaffolds

Recellularized dSMG scaffolds seeded with hMSCs or hSMG-SCs were harvested on day 7 for histological evaluation and on day 3 and day 7 for Ki67 immunofluorescence. Samples were fixed in 4% paraformaldehyde at 4 °C for 24 h, dehydrated in graded ethanol, embedded in paraffin and sectioned at 5 μm. Deparaffinized sections were stained with hematoxylin and eosin (H&E; Servicebio, China). For Ki67 staining, sections were deparaffinized, rehydrated and blocked with bovine serum albumin, followed by overnight incubation at 4 °C with an anti-Ki67 primary antibody (Proteintech, USA). After washing with PBS, sections were incubated with fluorophore-conjugated secondary antibodies and nuclei were counterstained with DAPI prior to imaging.

### 2.11 Scanning electron microscopy (SEM)

Native rat SMG, dSMG and recellularized scaffolds were fixed in 2.5% glutaraldehyde at 4 °C overnight. Small tissue fragments (approximately 2–3 mm) were rinsed in phosphate-buffered saline (PBS) and fixed in 2.5% glutaraldehyde at 4 °C overnight. For recellularized scaffolds, samples were collected after 7 days of culture, cut open with a sterile blade to expose the internal surface, and the cross-sections were fixed using the same procedure.

Following fixation, all specimens were washed three times with PBS and post-fixed in 1% osmium tetroxide for 1 h at room temperature. Samples were then dehydrated through a graded ethanol series (30%, 50%, 70%, 80%, 90%, and 100%, 10 min each), followed by critical-point drying (Quorum K850). Dried samples were mounted on aluminum stubs, sputter-coated with gold, and imaged using a scanning electron microscope (Hitachi SU8100, Tokyo, Japan) at an accelerating voltage of 5–10 kV.

### 2.12 Quantitative reverse transcription polymerase chain reaction (RT-qPCR)

RT-qPCR was used to evaluate gene expression in recellularized dSMG scaffolds seeded with hMSCs or hSMG-SCs. Constructs were harvested at day 3 and day 7, and total RNA was extracted using RNAzol® RT (MRC, USA). cDNA was synthesized using HiScript® III RT SuperMix Kit (Vazyme, China). Quantitative PCR was performed on a LightCycler 480 system (Roche, Switzerland) using ChamQ Universal SYBR qPCR Master Mix (Vazyme, China). GAPDH was used as the internal reference. For hMSC-seeded scaffolds, vimentin, COL1A1, MMP2 and CDH1 were analyzed. For hSMG-SC–seeded scaffolds, CK7, AMY1 and ACTA2 were analyzed. All measurements were performed in technical triplicate for each biological replicate, and primer sequences are provided in Supplementary Table S1.

### 2.13 Subcutaneous implantation in nude mice

Subcutaneous implantation was performed under inhalation anesthesia with isoflurane in nude mice using a gas anesthesia machine (Anhui Yaokun Biotechnology Co., Ltd., China). Anesthesia was induced with 5% isoflurane in oxygen delivered at 1 L/min and maintained with 3% isoflurane in oxygen delivered at 0.3 L/min. The route of administration was inhalation. During surgery, anesthetic depth was monitored by assessing respiration, muscle tone, and pedal withdrawal reflex. Subcutaneous implantation was performed using two experimental groups: the recellularized dSMG implant group and the acellular dSMG implant group. For the recellularized dSMG implant group, three decellularized submandibular gland scaffolds (dSMG) seeded with human submandibular gland epithelial stem cells (hSMG-SCs) were implanted (one implant per mouse; n = 3 nude mice). For the acellular dSMG implant group, three unseeded dSMG scaffolds were implanted (one implant per mouse; n = 3 nude mice).

All implants were placed subcutaneously in nude mice under aseptic conditions. Recellularized dSMG implants were implanted after 3 days of *in vitro* culture following cell seeding. At 8 weeks post-implantation, the implants together with surrounding tissues were harvested for gross inspection and histological analyses.

### 2.14 *In vivo* histology and immunostaining of implanted constructs

After subcutaneous implantation, explants from the recellularized and acellular groups were harvested with surrounding tissues, fixed in 4% paraformaldehyde at 4 °C for 24 h, dehydrated in graded ethanol, embedded in paraffin and sectioned at 5 μm. Deparaffinized sections were stained with hematoxylin and eosin (H&E; Servicebio, China). Human native SMG tissue and dSMG was processed in parallel for staining as a reference.

For CK7 and AQP5 immunohistochemistry, sections were deparaffinized and rehydrated, blocked with 5% bovine serum albumin for 30 min, and incubated overnight at 4 °C with an anti-CK7 or anti-AQP5 primary antibody (Proteintech, USA). Sections were then incubated with an HRP-conjugated secondary antibody and developed with DAB.

For immunofluorescence staining, sections were deparaffinized, rehydrated and blocked with 1% bovine serum albumin for 30 min, followed by overnight incubation at 4 °C with primary antibodies against CD31 or HLA (Proteintech, USA). After washing with PBS, sections were incubated with CoraLite®594-conjugated and CoraLite®488-conjugated secondary antibodies (Proteintech, USA), respectively. Nuclei were counterstained with DAPI prior to imaging.

### 2.15 Statistical analysis

Data are presented as mean ± SD. Unless otherwise stated, n represents biological replicates, including independent tissue samples, independently prepared scaffolds, or individual animals. For assays performed in technical triplicate, technical replicates were averaged before statistical analysis. Normality was assessed using the Shapiro–Wilk test. For normally distributed data, two-group comparisons were performed using unpaired two-tailed Student’s t-test, and comparisons among more than two groups were performed using one-way ANOVA followed by Tukey’s multiple-comparisons test. For data that did not meet normality assumptions, nonparametric tests were applied as appropriate. Statistical analysis was performed using GraphPad Prism 9 (GraphPad Software, USA). P < 0.05 was considered statistically significant.

## 3 Results

### 3.1 Preparation and characterization of decellularized rat SMG scaffolds

**FIGURE 1:**
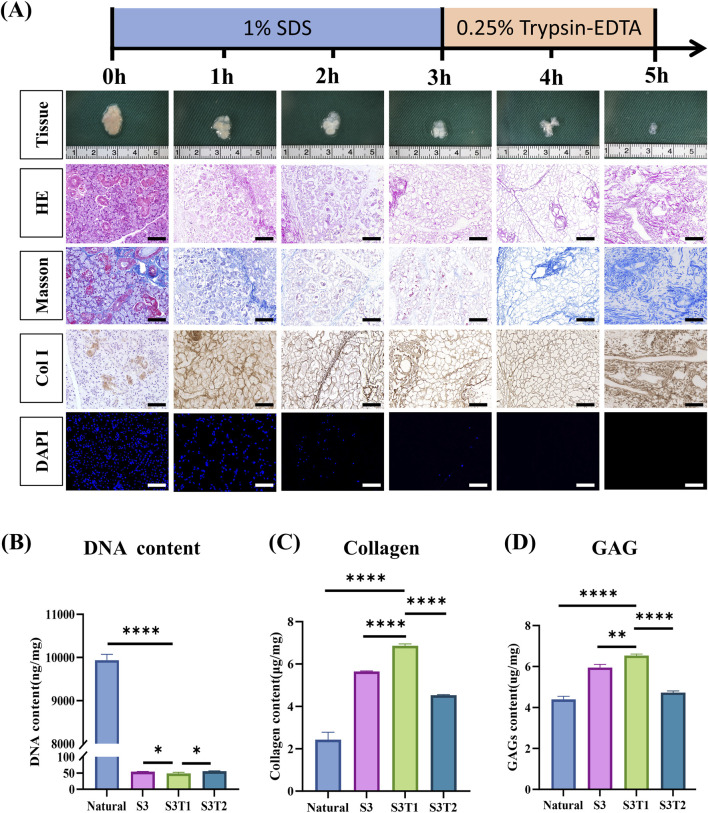
Preparation and characterization of decellularized rat SMG scaffolds. **(A)** Time-course decellularization of rat submandibular glands using 1% SDS followed by 0.25% trypsin–EDTA, showing macroscopic appearance and corresponding H&E, Masson’s trichrome, collagen I, and DAPI staining to illustrate progressive cell removal and ECM preservation. Scale bars: 100 μm. **(B)** Quantification of DNA content in native and decellularized SMGs. **(C)** Collagen content in native SMGs and decellularized scaffolds. **(D)** GAG content in native SMGs and decellularized scaffolds. Data are presented as mean ± SD (n = 3). ****p < 0.0001. SMG, submandibular gland; SDS, sodium dodecyl sulfate; trypsin–EDTA, trypsin–ethylenediaminetetraacetic acid; H&E, hematoxylin and eosin; DAPI, 4′,6-diamidino-2-phenylindole; ECM, extracellular matrix; GAG, glycosaminoglycan.

Rat submandibular glands were decellularized by three freeze-thaw cycles (liquid nitrogen-37 °C), followed by stepwise treatment with 1% SDS and 0.25% trypsin-EDTA under agitation at 200 rpm (Figure 1A). Macroscopically, the glands gradually changed from a pink, opaque appearance to a pale and translucent structure, indicating progressive cellular removal. Histological evaluation demonstrated that all protocols with a total processing time of 3 h were insufficient to achieve complete decellularization, as residual cellular and nuclear components were consistently observed by H&E staining, regardless of detergent or enzyme sequence (Supplementary Figure S1). In contrast, among the extended SDS-trypsin protocols, the S3T1 condition achieved effective nuclear clearance while preserving overall tissue architecture, whereas excessive trypsin exposure (S3T2) resulted in marked matrix disruption and tissue collapse (Figure 1A).

Consistently, Masson’s trichrome and collagen I staining revealed well-organized collagen fibers in the S3T1 group, whereas pronounced ECM fragmentation was observed in the S3T2 group. DAPI staining further confirmed near-complete removal of nuclear material under the S3T1 condition, as also shown by whole-section and regional imaging (Supplementary Figure S2), with markedly reduced nuclear signal observed in both the central and peripheral regions of S3T1 compared with native SMG. α-Gal immunohistochemistry showed strong positivity in native SMG but markedly reduced staining in the S3T1 scaffold after decellularization, further supporting reduced retention of immunogenic xenogeneic epitopes (Supplementary Figure S3).

Biochemical analyses supported these observations. DNA content was reduced from 9937.57 ± 133.74 ng/mg in native SMG to below 100 ng/mg in all decellularized groups (Figure 1B), with the S3T1 group showing the lowest residual DNA level (48.79 ± 3.72 ng/mg). In addition, agarose gel electrophoresis showed that DNA from native SMG was mainly present around 1000 bp, whereas the residual DNA in dSMG was markedly reduced and predominantly distributed below 100 bp after decellularization (Supplementary Figure S4). In parallel, collagen and glycosaminoglycan (GAG) contents were highest in the S3T1 group (6.86 ± 0.10 μg/mg and 6.53 ± 0.08 μg/mg, respectively), whereas both components were reduced following excessive digestion in the S3T2 group (Figures 1C,D).

Overall, these results identify S3T1 as the optimal decellularization protocol, providing efficient cellular removal while preserving key ECM components and structural integrity. Accordingly, the S3T1 was selected for subsequent experiments, and the resulting dSMG scaffold was further characterized. Representative tensile stress–strain curves showed that the dSMG retained measurable mechanical integrity after decellularization, although its tensile behavior differed from that of native SMG (Supplementary Figure S5). Sequential PBS wash analysis showed that SDS concentration progressively decreased during washing and reached a very low level in the final wash fraction, supporting effective detergent removal under the applied washing conditions (Supplementary Figure S6; Cebotari et al., 2010). Endotoxin testing demonstrated a low endotoxin level in the final scaffold extract, 0.0925 ± 0.008 EU/mL, supporting the suitability of the washed dSMG scaffold for subsequent *in vivo* implantation (Supplementary Figure S7). Surface-seeding CCK-8 assays on washed dSMG scaffolds also showed progressively increased metabolic activity from day 1 to day 9 in both hMSC- and hSMG-SC-seeded groups relative to acellular controls (Supplementary Figure S8), supporting the cytocompatibility of the washed dSMG scaffold after decellularization.

### 3.2 Characterization of ECM components in dSMG

**FIGURE 2:**
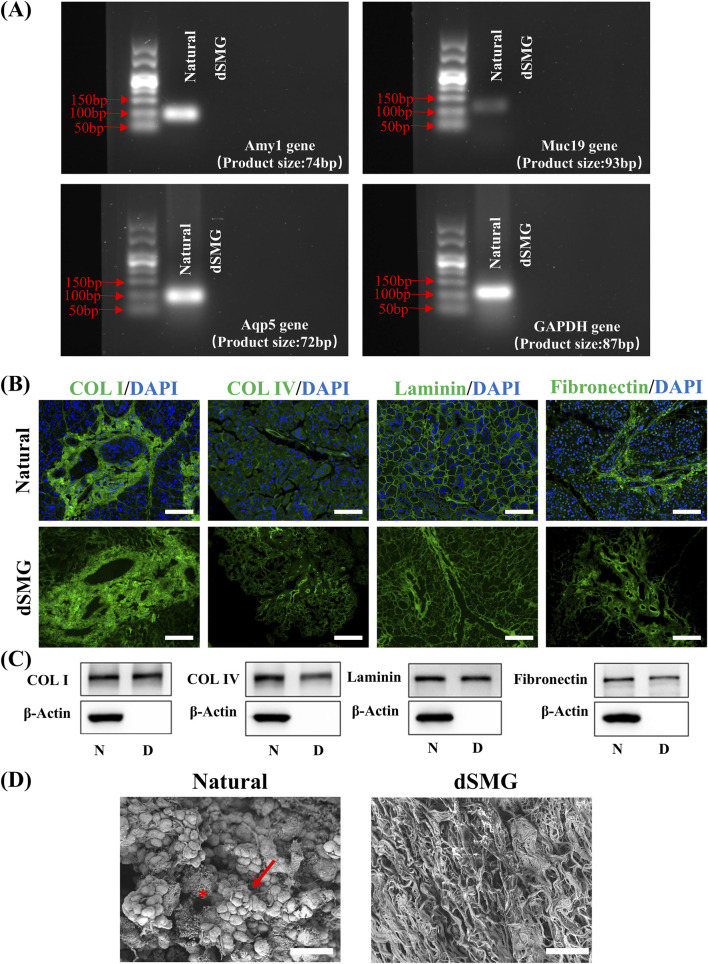
Characterization of ECM components in dSMG. **(A)** RT-PCR analysis of salivary gland-specific genes (Amy1, Muc19, Aqp5) and the housekeeping gene (GAPDH) in native SMGs and dSMGs. **(B)** Immunofluorescence staining for ECM proteins in native and decellularized SMGs. Collagen I, collagen IV, laminin, and fibronectin (green) were retained in dSMG, and nuclei were counterstained with DAPI (blue). Scale bar: 50 μm. **(C)** Western blot analysis of ECM proteins before (N) and after (D) decellularization. **(D)** Scanning electron micrographs showing surface morphology of native and decellularized SMG tissues. In the native SMG, the arrow indicates a acinar-like cluster, and the asterisk indicates a lumen-like space. Scale bar: 40 μm. ECM, extracellular matrix; dSMG, decellularized submandibular gland; RT-PCR, reverse transcription polymerase chain reaction; GAPDH, glyceraldehyde-3-phosphate dehydrogenase; DAPI, 4′,6-diamidino-2-phenylindole; N, native rat SMG; D, dSMG.

Decellularization efficacy and ECM preservation were assessed via RT-PCR, immunofluorescence, Western blotting, and SEM (Figure 2). RT-PCR showed loss of tissue-specific transcripts after decellularization, with Amy1, Muc19, Aqp5, and GAPDH detected only in native SMG (Figure 2A). Immunofluorescence staining showed that collagen I, collagen IV, laminin, and fibronectin were well retained in dSMG, with spatial patterns similar to native tissue and absence of nuclear signals (Figure 2B). Western blot analysis under equal total protein input showed retained signals for collagen I, collagen IV, laminin, and fibronectin in dSMG, while β-actin was markedly depleted after decellularization (Figure 2C; full-length blots are shown in Supplementary Figure S9, and densitometric analysis is shown in Supplementary Figure S10). SEM demonstrated a transition from well-organized acinar clusters in native SMG to a porous, fibrous architecture in dSMG, characteristic of a decellularized scaffold (Figure 2D).

### 3.3 Proteomic characterization of the dSMG

**FIGURE 3:**
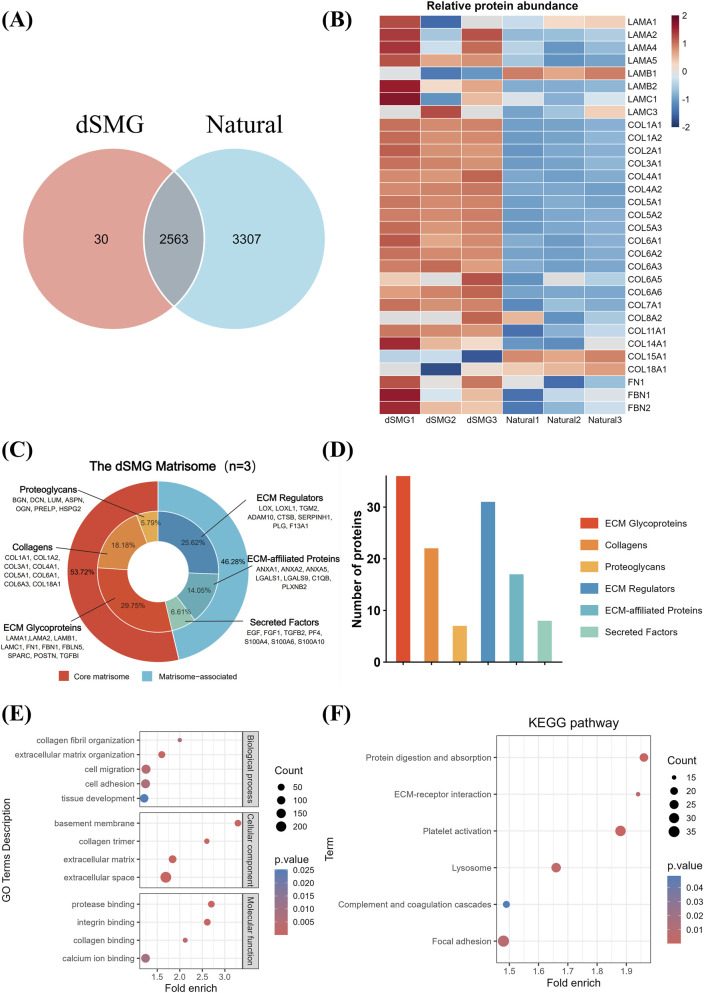
Proteomic profiling and functional enrichment of the dSMG extracellular matrix. **(A)** Venn diagram illustrating the total proteins identified in native SMG (5870) and dSMG (2593), with 2563 proteins shared between the two groups. **(B)** Heatmap showing relative abundance of ECM-related proteins, demonstrating preferential retention of structural ECM components in dSMG. **(C)** Classification of the dSMG matrisome into six subcategories according to the Matrisome database, with representative proteins listed. **(D)** Quantitative distribution of ECM protein categories. **(E)** GO analysis of dSMG proteins, highlighting enriched biological processes, cellular components, and molecular functions. **(F)** KEGG enrichment analysis of dSMG proteins. Proteomic analysis was performed using three biological replicates per group (n = 3). dSMG, decellularized submandibular gland; ECM, extracellular matrix; GO, Gene Ontology; KEGG, Kyoto Encyclopedia of Genes and Genomes.

Proteomic analysis identified 2,593 proteins in the dSMG and 5,870 in the native SMG, with 2,563 shared proteins, indicating substantial retention of matrix components after decellularization (Figure 3A). ECM-associated proteins, including laminins (LAMA1–5), collagens (COL1A1–COL6A3), and fibronectin (FN1), were relatively enriched in dSMG, as shown by heatmap analysis (Figure 3B). Quantitative ECM classification revealed that 121 ECM proteins were retained in dSMG, including 22 collagens (18.18%), 36 glycoproteins (29.75%), and 7 proteoglycans (5.79%) (Figures 3C,D). Most major structural collagens (e.g., COL1A1, COL1A2, COL4A1–2) present in native SMG were preserved, indicating effective retention of the fibrillar framework (Supplementary Figure S11). Matrisome-associated components such as 31 ECM regulators (25.62%), 17 affiliated proteins (14.05%), and 8 secreted factors (6.61%) were also maintained, preserving functional molecules like LOX, SERPINH1, and TGFB2. Comparison with the native SMG showed that while the total number of ECM proteins decreased post-decellularization, the relative proportions of each category remained comparable, suggesting compositional fidelity (Supplementary Figure S12). The overlapping ECM proteins between native and dSMG exhibited similar category distribution, further supporting preservation of both structural and signaling elements (Supplementary Figure S12). Biological replicates demonstrated consistent protein counts across all ECM subtypes, confirming the reproducibility of the decellularization process (Supplementary Figure S13). GO enrichment analysis showed that retained ECM proteins were mainly involved in matrix organization, collagen fibril assembly, and cell adhesion, while KEGG analysis highlighted enrichment in ECM–receptor interaction and focal adhesion pathways (Figures 3E,F). These findings confirm that the dSMG scaffold maintains critical structural and signaling components of the native ECM, supporting its potential for tissue remodeling and regeneration.

### 3.4 Recellularization of dSMG scaffolds with hMSCs and hSMG-SCs *in vitro*

To evaluate whether the decellularized submandibular gland scaffold could support intraductal cell delivery and subsequent recellularization, hMSCs and hSMG-SCs were independently introduced into the dSMG via the main duct and maintained under dynamic *in vitro* culture.

Prior to seeding, hMSCs exhibited a typical spindle-shaped morphology at passage 3 (Supplementary Figure S14) and displayed a mesenchymal immunophenotype, with high expression of CD73 (95.23%), CD90 (92.81%), and CD105 (92.02%) and low expression of hematopoietic markers CD45 (0.21%) and CD34 (0.24%) (Supplementary Figure S15). Immunofluorescence staining further confirmed expression of CD29, CD90, Vimentin, and HLA, verifying both mesenchymal identity and human origin (Supplementary Figure S16). In parallel, hSMG-SCs showed an epithelial-like morphology at passage 3 (Supplementary Figure S17). Flow cytometric analysis demonstrated robust expression of epithelial-associated markers, including EpCAM (99.8%), CD49f (99.8%), CD29 (100%), CD44 (92.9%), CD166 (100%), and HLA-ABC (99.9%), with absence of CD105 (2.26%) and CD45 (2.96%) expression (Supplementary Figure S18). Immunofluorescence staining further demonstrated positive expression of CK5, CK14, SOX2, EpCAM, and HLA, supporting a basal/ductal progenitor-like phenotype of hSMG-SCs (Supplementary Figure S19).

**FIGURE 4:**
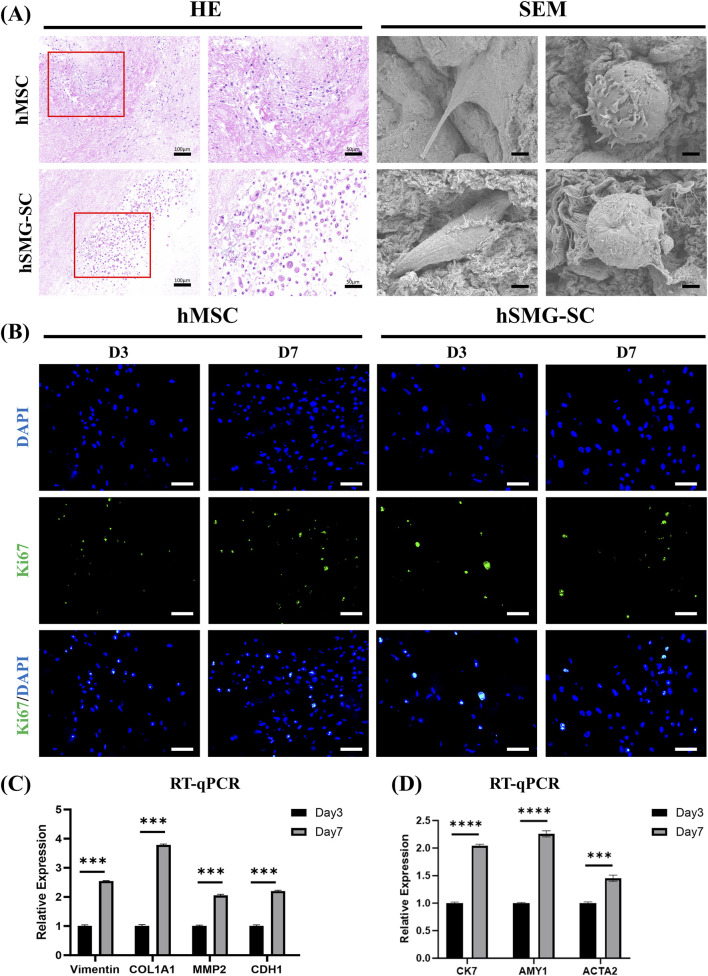
Recellularization of dSMG scaffolds with hMSCs and hSMG-SCs *in vitro*. **(A)** Histological and ultrastructural evaluation of dSMG scaffolds recellularized with hMSCs or hSMG-SCs after 7 days of dynamic culture. H&E staining shows cellular distribution and infiltration within the scaffold, with red boxes indicating regions shown at higher magnification. SEM images reveal representative stages of cell–matrix interaction within the dSMG scaffold, showing cells in flattened spreading and filopodia-rich attachment states, reflecting active adhesion and migration on the extracellular matrix. Scale bars: H&E low magnification, 100 μm; H&E high magnification, 50 μm; SEM, 2 μm. **(B)** Immunofluorescence staining for Ki67 in hMSC- and hSMG-SC–recellularized dSMG scaffolds at day 3 (D3) and day 7 (D7). Nuclei were counterstained with DAPI. Scale bar: 50 μm. **(C)** RT-qPCR analysis of mesenchymal identity and extracellular matrix remodeling–related genes (Vimentin, COL1A1, MMP2, and CDH1) in hMSC-recellularized dSMG scaffolds at day 3 and day 7. Gene expression levels were normalized to day 3. Data are presented as mean ± SD (n = 3). ***p < 0.001. **(D)** RT-qPCR analysis of CK7 and AMY1 and ACTA2 in hSMG-SC–recellularized dSMG scaffolds at day 3 and day 7. Relative expression levels were normalized to day 3. Data are presented as mean ± SD (n = 3). ***p < 0.001, ****p < 0.0001. dSMG, decellularized submandibular gland; hMSCs, human mesenchymal stem cells; hSMG-SCs, human submandibular gland epithelial stem cells; H&E, hematoxylin and eosin; SEM, scanning electron microscopy; DAPI, 4′,6-diamidino-2-phenylindole; D3, day 3; D7, day 7; RT-qPCR, reverse transcription quantitative polymerase chain reaction; CK7, cytokeratin 7; AMY1, amylase 1; ACTA2, actin alpha 2, smooth muscle; COL1A1, collagen type I alpha 1 chain; MMP2, matrix metallopeptidase 2; CDH1, cadherin 1.

Following intraductal injection, early cell retention within the dSMG scaffold was evaluated by DNA quantification at 4 h and 24 h after seeding (Supplementary Figure S20). After subtraction of scaffold background DNA, both hMSCs and hSMG-SCs showed detectable retained cell-associated DNA within the scaffold at both time points. After 7 days of dynamic culture, H&E staining revealed cellular distribution and infiltration throughout the matrix (Figure 4A). Scanning electron microscopy further demonstrated intimate cell–matrix interactions in both groups, with representative cells exhibiting either flattened spreading morphology or filopodia-rich attachment states, reflecting adhesion, migration, and adaptation to the dSMG extracellular matrix (Figure 4A).

Cellular proliferation within the scaffold was assessed by Ki67 immunofluorescence and metabolic assays. Both hMSC- and hSMG-SC–recellularized dSMG exhibited Ki67-positive cells at day 3 and day 7, suggesting ongoing cellular proliferation within the scaffold (Figure 4B). Consistently, CCK-8 assays showed progressively increased metabolic activity in recellularized constructs compared with acellular dSMG controls for both hMSCs and hSMG-SCs (Supplementary Figure S21).

At the transcriptional level, hMSC-recellularized dSMG exhibited significant upregulation of genes associated with mesenchymal identity and extracellular matrix remodeling, including Vimentin, COL1A1, MMP2, and CDH1, at day 7 relative to day 3 (Figure 4C). Interestingly, CDH1 (encoding E-cadherin) was also increased by day 7, which may reflect enhanced cell–cell contacts of MSCs within the 3D dSMG microenvironment. This gene expression profile suggests that the dSMG scaffold provides a bioactive niche that facilitates cell–matrix interaction and scaffold remodeling. In parallel, hSMG-SC–recellularized dSMG showed increased expression of salivary gland–related markers. RT-qPCR showed increased CK7, AMY1, and ACTA2 expression at day 7 compared with day 3 (Figure 4D), indicating induction of epithelial and gland-associated transcripts alongside a contractile marker within the dSMG microenvironment.

These results demonstrate that the dSMG scaffold supports intraductal delivery, retention, proliferation, and phenotype-associated gene expression of both hMSC and hSMG-SC, underscoring its suitability as a versatile biomimetic matrix for salivary gland tissue engineering.

### 3.5 *In vivo* integration and epithelial structure formation of recellularized dSMG scaffolds implants

**FIGURE 5:**
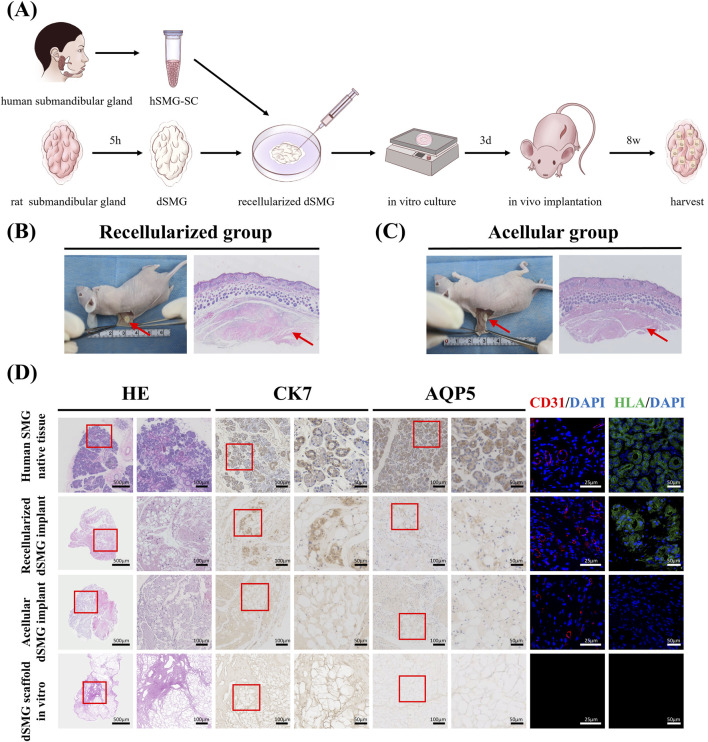
*In vivo* integration and remodeling of recellularized and acellular dSMG scaffolds. **(A)** Schematic illustration of the experimental workflow, including isolation of hSMG-SCs, preparation of dSMG scaffolds, recellularization, *in vitro* dynamic culture for 3 days, subcutaneous implantation in nude mice for 8 weeks, and subsequent harvest. **(B,C)** Gross appearance and H&E staining of nude mouse skin after subcutaneous implantation of recellularized dSMG implants **(B)** or acellular dSMG implants **(C)** for 8 weeks. Red arrows indicate the implanted scaffolds beneath the skin. Scale bars: 500 μm. **(D)** Histological and immunostaining characterization of human SMG native tissue, recellularized dSMG implant, acellular dSMG implant, and dSMG scaffold *in vitro* (pre-implantation). H&E staining shows overall tissue morphology; CK7 immunostaining identifies ductal or epithelial structures; AQP5 immunostaining evaluates acinar-associated differentiation; CD31/DAPI immunofluorescence indicates neovascularization; HLA/DAPI immunofluorescence indicates persistence of human cell–derived components. Red boxes denote regions shown at higher magnification. Scale bars: H&E low magnification, 500 μm; H&E high magnification, 100 μm; CK7 low magnification, 100 μm; CK7 high magnification, 50 μm; CD31/DAPI, 25 μm; HLA/DAPI, 50 μm. dSMG, decellularized submandibular gland; hSMG-SCs, human submandibular gland epithelial stem cells; H&E, hematoxylin and eosin; CK7, cytokeratin 7; CD31, platelet endothelial cell adhesion molecule-1; HLA, human leukocyte antigen; DAPI, 4′,6-diamidino-2-phenylindole.

To evaluate *in vivo* integration and remodeling of the dSMG scaffold, human submandibular gland epithelial stem cells (hSMG-SCs) were injected into the decellularized matrix via the main duct, followed by dynamic *in vitro* culture for 3 days prior to subcutaneous implantation in nude mice for 8 weeks (Figure 5A). The surgical procedure is shown in Supplementary Figure S22. Acellular dSMG implants without cell seeding served as controls.

Gross examination at explantation showed that both recellularized dSMG implants and acellular dSMG implants remained readily identifiable beneath the skin, with no obvious necrosis (Figures 5B,C). H&E staining of the overlying skin further supported stable implant retention in both groups (Figures 5B,C). Representative H&E images and semi-quantitative scoring showed mild-to-moderate inflammatory cell infiltration at the host–implant interface in both groups, with no evidence of a severe local inflammatory response (Supplementary Figure S23; Supplementary Table S2).

Histological analyses revealed clear differences among groups (Figure 5D). Human SMG native tissue displayed well-organized acinar and ductal structures and served as a reference. In the recellularized dSMG implant group, H&E staining showed epithelial structures with duct-like morphology within the scaffold, whereas such structures were not observed in the acellular dSMG implant group or in the dSMG scaffold *in vitro* prior to implantation. Consistently, cytokeratin 7 (CK7) immunostaining demonstrated distinct CK7-positive epithelial lining in recellularized dSMG implants, while CK7 staining was minimal or absent in acellular dSMG implants. To further assess acinar differentiation, AQP5 immunohistochemistry was performed. Strong AQP5 staining was present in native human SMG tissue, whereas only weak and focal AQP5-positive signals were detected in recellularized dSMG implants. In contrast, little to no AQP5 staining was observed in acellular dSMG implants or in dSMG scaffolds maintained *in vitro* before implantation. Together, these findings indicate that the recellularized dSMG scaffold supported epithelial organization and limited early acinar-associated differentiation *in vivo*, but did not achieve mature acinar reconstruction under the current implantation conditions.

Overall, these results indicate that pre-seeding dSMG with hSMG-SCs supports *in vivo* scaffold integration, promotes vascular ingrowth, and facilitates the formation of duct-like epithelial structures, together with limited AQP5-positive acinar-associated differentiation. These findings support early tissue organization *in vivo*, while indicating that full acinar maturation remains limited in the heterotopic subcutaneous model.

## 4 Discussion

Decellularized extracellular matrix (dECM) scaffolds are widely valued in regenerative medicine because they reduce immunogenicity while preserving organ-specific biochemical and structural cues that are difficult to replicate with synthetic or single-component materials(Kasravi et al., 2023; Zhang et al., 2022).By removing cellular antigens yet retaining collagens, laminins, fibronectin, proteoglycans, and matrix-bound signaling, dECM provides an instructive three-dimensional niche that supports cell adhesion, migration, survival, and tissue-relevant phenotype maintenance(Liu et al., 2025; Rijal, 2017).This native-like microenvironment enables constructive host remodeling such as vascular ingrowth and integration rather than inert fibrotic encapsulation(Aamodt and Grainger, 2016; Ostadi et al., 2024; Wang et al., 2023). Clinically and translationally, this rationale is supported by the widespread use of acellular dermal matrix and small intestinal submucosa–derived extracellular matrix scaffolds in reconstructive and soft-tissue repair surgeries(Da et al., 2021; Jin et al., 2025; Morrison and Hussey, 2006).Moreover, whole-organ decellularization studies in heart, lung, and kidney demonstrate that complex architectures can be preserved to support recellularization and achieve partial functional recovery (Barbulescu et al., 2022; Golebiowska et al., 2024).

Decellularization strategies are commonly categorized into physical, chemical, and enzymatic approaches (Moffat et al., 2022; Zhang et al., 2022). Physical methods such as freeze–thaw cycles, ultrasound, and high hydrostatic pressure disrupt cell membranes and enhance reagent penetration, but alone they often result in incomplete decellularization, especially in dense tissues (Rabbani et al., 2021). Chemical detergents remain the most effective means of cellular removal; ionic agents such as SDS achieve thorough decellularization but can disrupt ECM and deplete GAGs and proteins, whereas milder agents such as Triton X-100 better preserve structure but may leave residual debris (Li et al., 2018; Xu et al., 2017). Enzymatic agents such as trypsin and DNase can complement chemical methods but may weaken the ECM if overused (Barbulescu et al., 2022). Accordingly, detergent-based or combined chemical–enzymatic protocols are most commonly used for glandular tissue decellularization. For example, SDS-based treatments, typically 0.25%–0.5% for 12–48 h, effectively remove cellular and nuclear components while preserving extracellular matrix integrity and bioactivity in mammary and salivary gland scaffolds (Albusaily et al., 2025; Chissico Júnior et al., 2025). Perfusion-based liver decellularization typically uses sequential 1% SDS and 1% Triton X-100 for 48–96 h to clear parenchymal cells while retaining vasculature (Nari et al., 2013). Building on these observations, we developed an accelerated S3T1 workflow that combines physical, chemical, and enzymatic steps to shorten processing time while preserving ECM bioactivity. Briefly, S3T1 includes freeze–thaw pretreatment, 1% SDS for 3 h, 0.25% trypsin–EDTA for 1 h, and PBS washing to remove residual reagents; all steps were performed under continuous agitation at 200 rpm to enhance mass transport and promote more uniform decellularization throughout the gland. With this agitation-assisted and time-limited design, SDS plus trypsin–EDTA exposure totaled 4 h, and the overall detergent/enzyme processing time, including intermediate washes, was about 5 h. This workflow is substantially shorter than many conventional protocols and was designed to reduce detergent exposure while maintaining matrix integrity.

Our proteomic profiling showed that the salivary gland decellularized scaffold (dSMG) generated by the S3T1 method retained a functionally relevant subset of the native matrisome, including key structural and regulatory ECM proteins associated with cell adhesion and tissue regeneration. Major collagens, laminin subunits, fibronectin, and proteoglycans were preserved together with matrisome-associated regulators involved in ECM turnover and growth-factor binding (Diedrich et al., 2024). This pattern is consistent with reports from liver, kidney, and dental pulp ECM, in which retention of tissue-specific ECM proteins helps preserve bioactivity and supports cell adhesion and proliferation (Nowwarote et al., 2024; Park et al., 2025). Functional enrichment analysis further indicated that the retained proteins were mainly involved in extracellular matrix organization, collagen fibril assembly, ECM–receptor interaction, and focal adhesion signaling, pathways that regulate integrin-mediated adhesion, cytoskeletal organization, and cell survival (Knipper et al., 2015; Musiime et al., 2024; Wen et al., 2025). Previous studies have shown that collagen VI, fibronectin, and laminin can activate FAK and MAPK signaling, thereby promoting epithelial proliferation and constructive remodeling (Lin et al., 2023; Wishart et al., 2020). Together, these findings suggest that dSMG preserves matrix-derived biochemical cues relevant to cell–matrix interaction and early tissue organization. Similar proteomic studies in other organ-derived ECMs have also emphasized the importance of tissue-specific composition in supporting appropriate cell behavior and lineage-related differentiation (Zhang et al., 2024; Shi et al., 2023). Overall, our data indicate that dSMG provides a bioactive matrix environment relevant to salivary gland tissue engineering.

The *in vivo* fate and structural stability of a decellularized scaffold are important determinants of its ability to function as a durable regenerative template rather than a transient filler. In our study, both acellular and cell-seeded dSMG scaffolds remained clearly identifiable at the implantation site 8 weeks after transplantation, without obvious volume loss or collapse. Recellularized constructs also showed host vascular ingrowth and the formation of CK7-positive duct-like epithelial structures, indicating that the dSMG scaffold provided a stable microenvironment that supported cell survival and tissue remodeling. Together, these findings support *in vivo* persistence, host integration, and early epithelial organization of dSMG over the 8-week implantation period.

Several limitations should be acknowledged. First, the *in vivo* study used a heterotopic subcutaneous implantation model in immunodeficient mice. This model is suitable for evaluating scaffold persistence, host tolerance, vascularization, and early epithelial organization, but it does not reproduce the orthotopic salivary gland niche, including ductal connection, saliva flow, parasympathetic innervation, and gland-specific epithelial–mesenchymal interactions. Accordingly, functional outcomes such as saliva production and composition were not assessed, and the *in vivo* findings should be interpreted as evidence of biocompatibility and partial tissue remodeling rather than definitive functional gland regeneration. Second, although duct-like epithelial organization and vascular ingrowth were observed, reconstruction of a functional gland will likely require coordinated restoration of acinar, myoepithelial, endothelial, and neural components. Future studies should therefore test co-seeding or sequential seeding strategies together with orthotopic implantation in a gland injury model. Third, longer-term scaffold fate, degradation kinetics, and quantitative mechanical changes were not systematically characterized and may influence epithelial maturation and remodeling over time. In addition, the present study did not include a direct side-by-side comparison with conventional longer decellularization protocols, and DNA fragment size was assessed qualitatively rather than by precise fragment-length quantification. These issues should be addressed in future work to determine whether this dSMG platform can progress from early structural organization toward durable functional restoration.

Overall, the present study shows that dSMG can support human cell retention, proliferation, and early epithelial organization *in vivo*. However, the limited AQP5 signal and the heterotopic implantation model indicate that this platform currently supports early remodeling rather than full functional gland regeneration.

## 5 Conclusion

In conclusion, we developed a rapidly generated decellularized submandibular gland scaffold using the S3T1 protocol, with efficient decellularization and preservation of key matrix components under the conditions tested. The resulting dSMG supported intraductal recellularization with hMSCs and hSMG-SCs, as well as vascular ingrowth and duct-like epithelial organization *in vivo*. However, acinar-associated differentiation remained limited. These findings support dSMG as an ECM-based platform for salivary gland tissue engineering, while further orthotopic studies are needed to evaluate long-term remodeling and functional recovery.

## Data Availability

The mass spectrometry proteomics data presented in the study have been deposited to the ProteomeXchange Consortium via the PRIDE partner repository with the dataset identifier PXD077315. Other raw data supporting the conclusions of this article are included in the article and its Supplementary Material.

## References

[B1] Aamodt J. M. Grainger D. W. (2016). Extracellular matrix-based biomaterial scaffolds and the host response. Biomaterials 86, 68–82. 10.1016/j.biomaterials.2016.02.003 26890039 PMC4785021

[B2] Albusaily N. S. Alotaibi D. H. Jasser R. A. Alsarhan M. Aloraini S. Koppolu P. (2025). Decellularization of rat submandibular gland for salivary gland tissue-engineering applications. Int. Dent. J. 75, 1176–1182. 10.1016/j.identj.2024.07.1209 39112112 PMC11976612

[B3] Barbulescu G. I. Bojin F. M. Ordodi V. L. Goje I. D. Barbulescu A. S. Paunescu V. (2022). Decellularized extracellular matrix scaffolds for cardiovascular tissue engineering: current techniques and challenges. Int. J. Mol. Sci. 23, 13040. 10.3390/ijms232113040 36361824 PMC9658138

[B5] Brown M. Li J. Moraes C. Tabrizian M. Li-Jessen N. Y. K. (2022). Decellularized extracellular matrix: new promising and challenging biomaterials for regenerative medicine. Biomaterials 289, 121786. 10.1016/j.biomaterials.2022.121786 36116171

[B7] Cebotari S. Tudorache I. Jaekel T. Hilfiker A. Dorfman S. Ternes W. (2010). Detergent decellularization of heart valves for tissue engineering: toxicological effects of residual detergents on human endothelial cells. Artif. Organs 34, 206–210. 10.1111/j.1525-1594.2009.00796.x 20447045

[B8] Chissico JúNIOR F. Santos Da Silva T. Vieira Meirelles F. Monzani P. S. Fornari Laurindo L. Maria Barbalho S. (2025). A review on bioengineering the bovine mammary gland: the role of the extracellular matrix and reconstruction prospects. Bioeng. (Basel) 12, 501. 10.3390/bioengineering12050501 40428120 PMC12108683

[B9] Crapo P. M. Gilbert T. W. Badylak S. F. (2011). An overview of tissue and whole organ decellularization processes. Biomaterials 32, 3233–3243. 10.1016/j.biomaterials.2011.01.057 21296410 PMC3084613

[B10] Da L. C. Huang Y. Z. Xie H. Q. Zheng B. H. Huang Y. C. Du S. R. (2021). Membranous extracellular matrix-based scaffolds for skin wound healing. Pharmaceutics 13, 1796. 10.3390/pharmaceutics13111796 34834211 PMC8620109

[B11] Demichev V. Messner C. B. Vernardis S. I. Lilley K. S. Ralser M. (2020). DIA-NN: neural networks and interference correction enable deep proteome coverage in high throughput. Nat. Methods 17, 41–44. 10.1038/s41592-019-0638-x 31768060 PMC6949130

[B12] Diedrich A. M. Daneshgar A. Tang P. Klein O. Mohr A. Onwuegbuchulam O. A. (2024). Proteomic analysis of decellularized mice liver and kidney extracellular matrices. J. Biol. Eng. 18, 17. 10.1186/s13036-024-00413-8 38389090 PMC10885605

[B13] Faulk D. M. Carruthers C. A. Warner H. J. Kramer C. R. Reing J. E. Zhang L. (2014). The effect of detergents on the basement membrane complex of a biologic scaffold material. Acta Biomater. 10, 183–193. 10.1016/j.actbio.2013.09.006 24055455 PMC3857635

[B14] Gao Z. Wu T. Xu J. Liu G. Xie Y. Zhang C. (2014). Generation of bioartificial salivary gland using whole-organ decellularized bioscaffold. Cells Tissues Organs 200, 171–180. 10.1159/000371873 25824480

[B15] Golebiowska A. A. Intravaia J. T. Sathe V. M. Kumbar S. G. Nukavarapu S. P. (2024). Decellularized extracellular matrix biomaterials for regenerative therapies: advances, challenges and clinical prospects. Bioact. Mater 32, 98–123. 10.1016/j.bioactmat.2023.09.017 37927899 PMC10622743

[B16] Guo W. Y. Wang W. H. Xu P. Y. Kankala R. K. Chen A. Z. (2024). Decellularised extracellular matrix-based injectable hydrogels for tissue engineering applications. Biomater. Transl. 5, 114–128. 10.12336/biomatertransl.2024.02.003 39351160 PMC11438603

[B17] He M. Callanan A. Lagaras K. Steele J. A. M. Stevens M. M. (2017). Optimization of SDS exposure on preservation of ECM characteristics in whole organ decellularization of rat kidneys. J. Biomed. Mater Res. B Appl. Biomater. 105, 1352–1360. 10.1002/jbm.b.33668 27062181

[B18] Jin C. Zhang X. Jin Y. Chien P. N. Heo C. Y. (2025). Acellular extracellular matrix scaffolds in regenerative medicine: advances in decellularization and clinical applications. J. Funct. Biomater. 16, 383. 10.3390/jfb16100383 41149729 PMC12565388

[B19] Kasravi M. Ahmadi A. Babajani A. Mazloomnejad R. Hatamnejad M. R. Shariatzadeh S. (2023). Immunogenicity of decellularized extracellular matrix scaffolds: a bottleneck in tissue engineering and regenerative medicine. Biomater. Res. 27, 10. 10.1186/s40824-023-00348-z 36759929 PMC9912640

[B20] Knipper J. A. Willenborg S. Brinckmann J. Bloch W. Maaß T. Wagener R. (2015). Interleukin-4 receptor α signaling in myeloid cells controls collagen fibril assembly in skin repair. Immunity 43, 803–816. 10.1016/j.immuni.2015.09.005 26474656 PMC4681399

[B21] Li N. Li Y. Gong D. Xia C. Liu X. Xu Z. (2018). Efficient decellularization for bovine pericardium with extracellular matrix preservation and good biocompatibility. Interact. Cardiovasc Thorac. Surg. 26, 768–776. 10.1093/icvts/ivx416 29340634

[B23] Li Y. Li X. Pang R. Yang G. Tian M. Zhao T. (2022). Diagnosis, prevention, and treatment of radiotherapy-induced xerostomia: a review. J. Oncol. 2022, 7802334. 10.1155/2022/7802334 36065305 PMC9440825

[B24] Lilliu M. A. Seo Y. J. Isola M. Charbonneau A. M. Zeitouni A. El-Hakim M. (2016). Natural extracellular matrix scaffolds recycled from human salivary digests: a morphometric study. Oral Dis. 22, 313–323. 10.1111/odi.12444 26785831

[B25] Lin S. Wang J. Mukherjee P. Mao R. West G. Czarnecki D. (2023). DOP32 Crohn’s disease stricture matrisome analysis reveals the anti-fibrotic activity of milk-fat globule-epidermal growth factor 8 (MFGE8). J. Crohn's Colitis 17, i96. 10.1093/ecco-jcc/jjac190.0072

[B26] Liu J. Song Q. Yin W. Li C. An N. Le Y. (2025). Bioactive scaffolds for tissue engineering: a review of decellularized extracellular matrix applications and innovations. Explor. (Beijing) 5, 20230078. 10.1002/EXP.20230078 40040827 PMC11875452

[B27] Lopera Higuita M. Shortreed N. A. Dasari S. Griffiths L. G. (2022). Basement membrane of tissue engineered extracellular matrix scaffolds modulates rapid human endothelial cell recellularization and promote quiescent behavior after monolayer formation. Front. Bioeng. Biotechnol. 10, 903907. 10.3389/fbioe.2022.903907 35983533 PMC9379346

[B28] Lynge Pedersen A. M. BelstrøM D. (2019). The role of natural salivary defences in maintaining a healthy oral microbiota. J. Dent. 80 (Suppl. 1), S3–s12. 10.1016/j.jdent.2018.08.010 30696553

[B29] Matsuoka M. Soria S. A. Pires J. R. Sant'Ana A. C. P. Freire M. (2025). Natural and induced immune responses in oral cavity and saliva. BMC Immunol. 26, 34. 10.1186/s12865-025-00713-8 40251519 PMC12007159

[B30] Moffat D. Ye K. Jin S. (2022). Decellularization for the retention of tissue niches. J. Tissue Eng. 13, 20417314221101151. 10.1177/20417314221101151 35620656 PMC9128068

[B31] Morrison W. A. Hussey A. J. (2006). Extracellular matrix as a bioactive material for soft tissue reconstruction. ANZ J. Surg. 76, 1047. 10.1111/j.1445-2197.2006.03970.x 17199686

[B32] Musiime M. Erusappan P. M. Cukierman E. Chang J. Molven A. Hansen U. (2024). Fibroblast integrin α11β1 is a collagen assembly receptor in mechanoregulated fibrillar adhesions. Matrix Biol. 134, 144–161. 10.1016/j.matbio.2024.10.006 39406317 PMC12965127

[B33] Naba A. (2023). Ten years of extracellular matrix proteomics: accomplishments, challenges, and future perspectives. Mol. Cell Proteomics 22, 100528. 10.1016/j.mcpro.2023.100528 36918099 PMC10152135

[B34] Nari G. A. Cid M. ComíN R. Reyna L. Juri G. Taborda R. (2013). Preparation of a three-dimensional extracellular matrix by decellularization of rabbit livers. Rev. Esp. Enferm. Dig. 105, 138–143. 10.4321/s1130-01082013000300004 23735020

[B35] Nascimento M. L. Farias A. B. Carvalho A. T. Albuquerque R. F. Ribeiro L. N. Leao J. C. (2019). Impact of xerostomia on the quality of life of patients submitted to head and neck radiotherapy. Med. Oral Patol. Oral Cir. Bucal 24, e770–e775. 10.4317/medoral.23131 31655838 PMC6901149

[B36] Nowwarote N. Chahlaoui Z. Petit S. Duong L. T. Dingli F. Loew D. (2024). Decellularized extracellular matrix derived from dental pulp stem cells promotes gingival fibroblast adhesion and migration. BMC Oral Health 24, 1166. 10.1186/s12903-024-04882-7 39354504 PMC11443845

[B37] Ostadi Y. Khanali J. Tehrani F. A. Yazdanpanah G. Bahrami S. Niazi F. (2024). Decellularized extracellular matrix scaffolds for soft tissue augmentation: from host-scaffold interactions to bottlenecks in clinical translation. Biomater. Res. 28, 0071. 10.34133/bmr.0071 39247652 PMC11378302

[B38] Park S. Lee M. J. Kim H. J. Choi S. Cho J. H. Lee S. (2025). Skin ECM provides a bio-derived platform for supporting dermal renewal and matrix synthesis. J. Microbiol. Biotechnol. 35, e2505015. 10.4014/jmb.2505.05015 40537912 PMC12197817

[B39] Poornejad N. Schaumann L. B. Buckmiller E. M. Momtahan N. Gassman J. R. Ma H. H. (2016). The impact of decellularization agents on renal tissue extracellular matrix. J. Biomater. Appl. 31, 521–533. 10.1177/0885328216656099 27312837

[B40] Rabbani M. Zakian N. Alimoradi N. (2021). Contribution of physical methods in decellularization of animal tissues. J. Med. Signals Sens. 11, 1–11. 10.4103/jmss.JMSS_2_20 34026585 PMC8043117

[B41] Rijal G. (2017). The decellularized extracellular matrix in regenerative medicine. Regen. Med. 12, 475–477. 10.2217/rme-2017-0046 28703695

[B42] Rose S. C. Larsen M. Xie Y. Sharfstein S. T. (2023). Salivary gland bioengineering. Bioeng. (Basel) 11, 28. 10.3390/bioengineering11010028 38247905 PMC10813147

[B43] Shao X. Gomez C. D. Kapoor N. Considine J. M. Grams C. Gao Y. T. (2023). MatrisomeDB 2.0: 2023 updates to the ECM-protein knowledge database. Nucleic Acids Res. 51, D1519–d1530. 10.1093/nar/gkac1009 36399478 PMC9825471

[B44] Shi Y. Wang Y. Shan Z. Gao Z. (2023). Decellularized rat submandibular gland as an alternative scaffold for dental pulp regeneration. Front. Bioeng. Biotechnol. 11, 1148532. 10.3389/fbioe.2023.1148532 37152652 PMC10160494

[B46] Song W. Liu H. Su Y. Zhao Q. Wang X. Cheng P. (2024). Current developments and opportunities of pluripotent stem cells-based therapies for salivary gland hypofunction. Front. Cell Dev. Biol. 12, 1346996. 10.3389/fcell.2024.1346996 38313227 PMC10834761

[B47] Wang T. Huang Q. Rao Z. Liu F. Su X. Zhai X. (2023). Injectable decellularized extracellular matrix hydrogel promotes salivary gland regeneration *via* endogenous stem cell recruitment and suppression of fibrogenesis. Acta Biomater. 169, 256–272. 10.1016/j.actbio.2023.08.003 37557943

[B48] Wen S. Y. Ng S. C. Noriega L. Chen T. J. Chen C. J. Lee S. D. (2025). Echinacoside promotes collagen synthesis and survival *via* activation of IGF-1 signaling to alleviate UVB-induced dermal fibroblast photoaging. Biofactors 51, e2152. 10.1002/biof.2152 39780317

[B49] Wishart A. L. Conner S. J. Guarin J. R. Fatherree J. P. Peng Y. Mcginn R. A. (2020). Decellularized extracellular matrix scaffolds identify full-length collagen VI as a driver of breast cancer cell invasion in obesity and metastasis. Sci. Adv. 6, eabc3175. 10.1126/sciadv.abc3175 33087348 PMC7577726

[B50] Xu K. Kuntz L. A. Foehr P. Kuempel K. Wagner A. Tuebel J. (2017). Efficient decellularization for tissue engineering of the tendon-bone interface with preservation of biomechanics. PLoS One 12, e0171577. 10.1371/journal.pone.0171577 28170430 PMC5295703

[B51] Yang H. Xia J. Qian Y. Gu X. Cong M. (2025). From production to the Clinic: Decellularized Extracellular Matrix as a biomaterial for tissue engineering and regenerative medicine. Bioeng. (Basel) 13, 24. 10.3390/bioengineering13010024 41595956 PMC12837877

[B52] Yoon Y. J. Kim D. Tak K. Y. Hwang S. Kim J. Sim N. S. (2022). Salivary gland organoid culture maintains distinct glandular properties of murine and human major salivary glands. Nat. Commun. 13, 3291. 10.1038/s41467-022-30934-z 35672412 PMC9174290

[B53] Zhang X. Chen X. Hong H. Hu R. Liu J. Liu C. (2022). Decellularized extracellular matrix scaffolds: recent trends and emerging strategies in tissue engineering. Bioact. Mater 10, 15–31. 10.1016/j.bioactmat.2021.09.014 34901526 PMC8637010

[B54] Zhang S. Guo Y. Lu Y. Liu F. Heng B. C. Deng X. (2024). The considerations on selecting the appropriate decellularized ECM for specific regeneration demands. Mater Today Bio 29, 101301. 10.1016/j.mtbio.2024.101301 39498148 PMC11532911

