# Supplementary material for: Rapid SDS/trypsin decellularization of rat submandibular gland yields an ECM scaffold supporting salivary gland tissue engineering
Source: Front Bioeng Biotechnol. 2026 Apr 28;14:1795289. doi: 10.3389/fbioe.2026.1795289 (PMC13161092; doi:10.3389/fbioe.2026.1795289)

Supplementary Material

Rapid SDS/trypsin decellularization of rat submandibular gland yields an ECM scaffold supporting salivary gland tissue regeneration

Jie Gao^#1,2^, Bo Kyoung Kang ^#1^, Xiuxia Wang ^1^, Minyan He ^1^, Zhaoqi Yuan ^1^, Shun Yu ^3^, Lin Lu ^1*^, Jun Yang ^1*^

^1^Department of Plastic and Reconstructive Surgery, Shanghai Ninth People's Hospital, Shanghai Jiao Tong University School of Medicine, Shanghai, People's Republic of China.

^2^Shanghai Institute for Plastic and Reconstructive Surgery, Shanghai Ninth People's Hospital, Shanghai Jiao Tong University School of Medicine, Shanghai, People's Republic of China.

^3^Department of Burns and Plastic Surgery, The Affiliated Hospital of Jiangnan University, Wuxi, China.

# Contributed equally.

*** Correspondence:**Lin Lu
[lulin_12345@163.com](mailto:lulin_12345@163.com)

Jun Yang
yj55569@hotmail.com

# Supplementary Table

**Supplementary Table 1 |** Primers of genes for RT-qPCR

| **Gene name** | **Forward primer** | **Reverse primer** |
| --- | --- | --- |
| rat Amy1 | GGA GAA GGT TGG GGT TTC AT | TGT CCT CGC TGG TTG TCA T |
| rat Aqp5 | CTG TCG TCA AAG GCA CAT A | CGA TGG TCT TCT TCC TCT C |
| rat Muc19 | CGA TGG GCT CTG CCA GTA | TCA CAG CAG GGG ACA CTC TC |
| rat GAPDH | TGT TCC TAC CCC CAA TGT AT | TTC ACC ACC TTC TTG ATG TC |
| human Vimentin | CTG TAA GTT GGT AGC ACT GAG | TTA GGG GAA ACC GTT AGA C |
| human COL1A1 | AGG GCC AAG ACG AAG ACA TC | GTC GGT GGG TGA CTC TGA GC |
| human MMP2 | CCC ATT TTG ATG ACG ATG A | TTG TAC TCC TTG CCA TTG AAC |
| human CDH1 | AAG ACA AAG AAG GCA AGG T | AGA GAG TGT ATG TGG CAA TG |
| human KRT7 | CGA GGA TAT TGC CAA CCG CAG | CCT CAA TCT CAG CCT GGA GCC |
| human AMY1 | GGG AAA GAT ACC AAC CAG TTA | ACT CAC AGC ATT ACC ACA CAT A |
| human ACTA2 | CGT GGC TAT TCC TTC GTT ACT A | ATC AGG CAA CTC GTA ACT CTT C |
| human GAPDH | GGG AAG GTG AAG GTC GGA GT | GGG GTC ATT GAT GGC AAC A |

# Supplementary materials and methods

## Flow cytometry

Human mesenchymal stem cells (hMSCs) and human submandibular gland epithelial stem cells (hSMG-SCs) were detached using 0.25% trypsin–EDTA, neutralized with complete medium, and washed twice with PBS containing 2% fetal bovine serum. Approximately 1 × 10^5 cells per sample were incubated at 4 °C for 30 min in the dark with fluorophore-conjugated antibodies (BioLegend, USA). For hMSCs, antibodies against CD73, CD90, CD105, CD34, and CD45 were used (BioLegend, USA). For hSMG-SCs, antibodies against EpCAM, CD49f, CD29, CD44, CD166, HLA-ABC, CD105, and CD45 were used (BioLegend, USA). Matched fluorophore-conjugated isotype controls were included (BioLegend, USA). After staining, cells were washed twice and analyzed on a CytoFLEX flow cytometer (Beckman Coulter, USA). At least 10,000 events were collected per sample. Debris and doublets were excluded by forward/side scatter and singlet gating. Data were analyzed using FlowJo software (Tree Star, USA).

## Mechanical testing

Mechanical properties of native SMG and decellularized SMG scaffolds, dSMG, were evaluated by uniaxial tensile testing. Rectangular specimens were prepared from native SMG and dSMG scaffolds and tested using a universal testing machine (Daixuan Biology, China). The initial gauge length was 5 mm, and the cross-sectional area was 0.5 mm². Samples were stretched at a displacement rate of 0.1667 mm/s until failure. Force–displacement curves were recorded, and representative tensile stress–strain curves were generated by converting force to stress and displacement to strain. Stress was calculated as force divided by cross-sectional area, and strain was calculated as displacement divided by the initial gauge length. Representative curves are shown in Supplementary Figure 5.

## Residual SDS assay

Residual SDS in wash solutions was quantified using an SDS detection kit (BioBioPha, China). During the 30 min PBS wash after 1% SDS treatment, wash fractions were collected every 10 min, yielding three samples, SDS-W1 to SDS-W3. During the subsequent 30 min PBS wash after trypsin treatment, three additional fractions were collected at 10 min intervals, Tryp-W1 to Tryp-W3. A 0.1% SDS stock solution was prepared by dissolving 0.01 g SDS in 10 mL distilled water, and serial standards of 0–0.014% were used to generate a standard curve. For each standard or appropriately diluted sample, 50 μL solution was mixed with 50 μL solution A and 1.5 mL solution B, vortexed for 3 min, and centrifuged at 5000 rpm for 5 min at room temperature. The upper phase was collected, and absorbance at 499 nm was measured with a spectrophotometer. SDS concentration was determined from the standard curve and multiplied by the dilution factor.

## Endotoxin assay

Endotoxin levels in the final scaffold extract were measured using a chromogenic Limulus Amebocyte Lysate assay kit (GenScript, USA). Before testing, each scaffold was incubated in 1 mL LAL Reagent Water for 24 h to prepare the extract. Endotoxin standards of 0.01, 0.025, 0.05, and 0.1 EU/mL were prepared to generate a standard curve. Briefly, 100 μL of standard or sample extract was mixed with reconstituted LAL, incubated at 37°C, reacted with chromogenic substrate, and terminated with the kit-provided stop and color-stabilizing solutions according to the manufacturer’s instructions. Absorbance was measured at 545 nm, and endotoxin concentration was calculated from the standard curve.

## DNA-based evaluation of early cell retention

To estimate early cell retention within dSMG scaffolds after intraductal delivery, hMSCs or hSMG-SCs were resuspended at 1 × 10⁷ cells/mL, and 100 μL of the cell suspension was injected into the main duct of each scaffold. At 4 h and 24 h after seeding, total DNA associated with the recellularized scaffolds was extracted using (TIANGEN, China) and quantified according to the manufacturer’s instructions. Acellular dSMG scaffolds processed in parallel at the corresponding time points were used to determine background scaffold DNA. Retained cell-associated DNA was calculated by subtracting the background DNA content of acellular scaffolds from the total DNA measured in recellularized scaffolds. Data are presented as mean ± SD, n = 3.

## CCK-8 Assay

Cell proliferation and metabolic activity were evaluated using a Cell Counting Kit-8 assay. For internal recellularization, dSMG scaffolds injected with hMSCs or hSMG-SCs were cultured in 24-well plates, and for surface-seeding assays, dSMG scaffolds were placed in 48-well plates to cover the well bottom and seeded with hMSCs or hSMG-SCs on the scaffold surface. Acellular dSMG scaffolds served as controls. At days 1, 3, 5, 7, and 9, the medium was replaced with CCK-8 reaction solution and incubated at 37 °C for 1 h with gentle shaking at 50 rpm. For 24-well plates, 900 μL of fresh complete medium and 100 μL of CCK-8 working solution were added to each well. For 48-well plates, 360 μL of fresh complete medium and 40 μL of CCK-8 working solution were added to each well. After incubation, 100 μL of the supernatant was transferred to a 96-well plate (Corning, USA), and absorbance at 450 nm was measured using a microplate reader (BioTek, USA). Acellular dSMG scaffolds processed in parallel were used for background subtraction. Net absorbance values were used to quantify cell proliferation over time. Data are presented as mean ± SD, n = 3.

# Supplementary figures


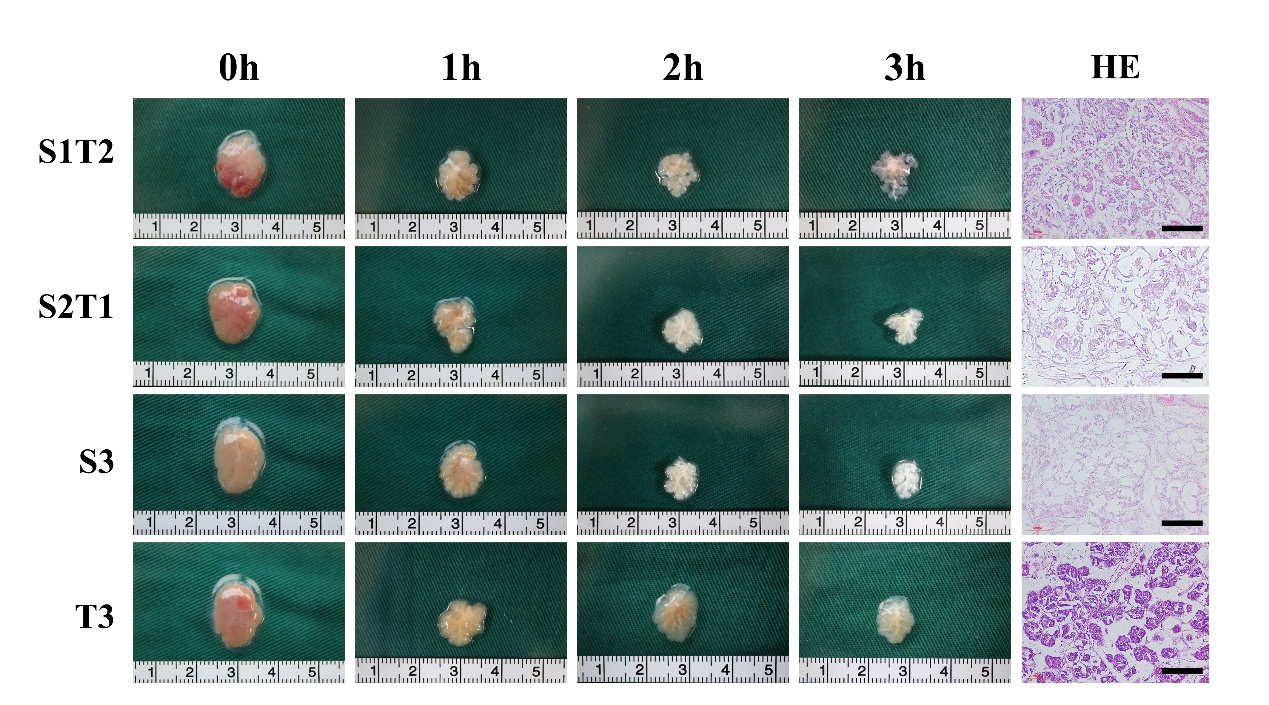


**Supplementary Figure 1.** **Gross appearance and histological evaluation of rat submandibular glands during decellularization using different protocols.** Representative macroscopic images were acquired at 0, 1, 2, and 3 h for each group. S indicates treatment with 1% SDS, and T indicates treatment with 0.25% Trypsin–EDTA. Corresponding H&E-stained sections are shown in the rightmost column. Histological examination revealed the presence of residual cellular components in all four groups, indicating incomplete decellularization under these conditions. Scale bar = 100 μm.


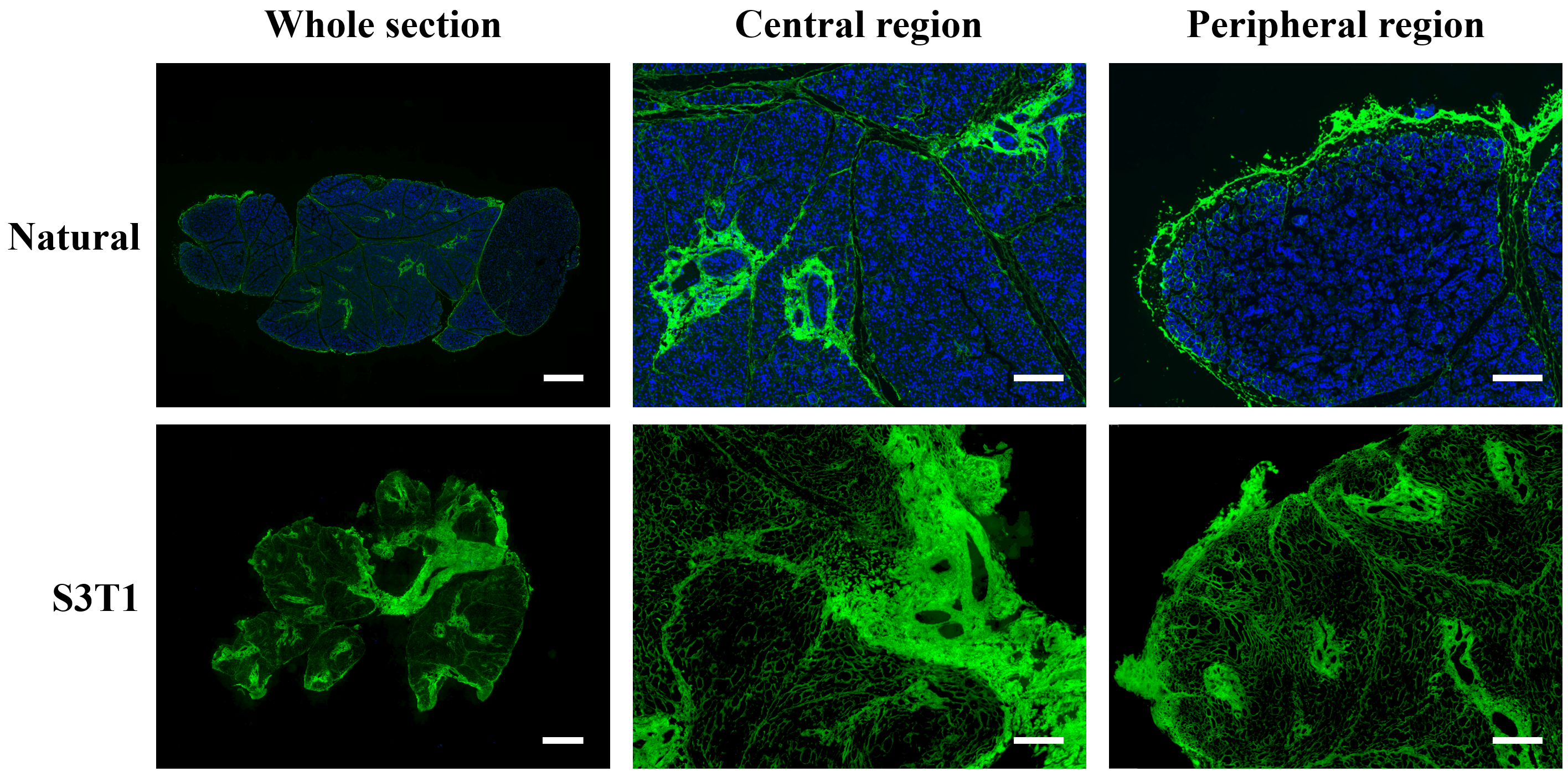


**Supplementary Figure 2. Whole-section and regional immunofluorescence images of native SMG and S3T1 scaffolds stained for COL I (green) and DAPI (blue).** COL I staining outlines the overall gland architecture, while DAPI indicates nuclear material. Compared with native SMG, S3T1 showed markedly reduced nuclear signal in both the central and peripheral regions, consistent with a homogeneous decellularization pattern throughout the gland section. Scale bars, 1 mm for whole-section images and 200 μm for regional images.


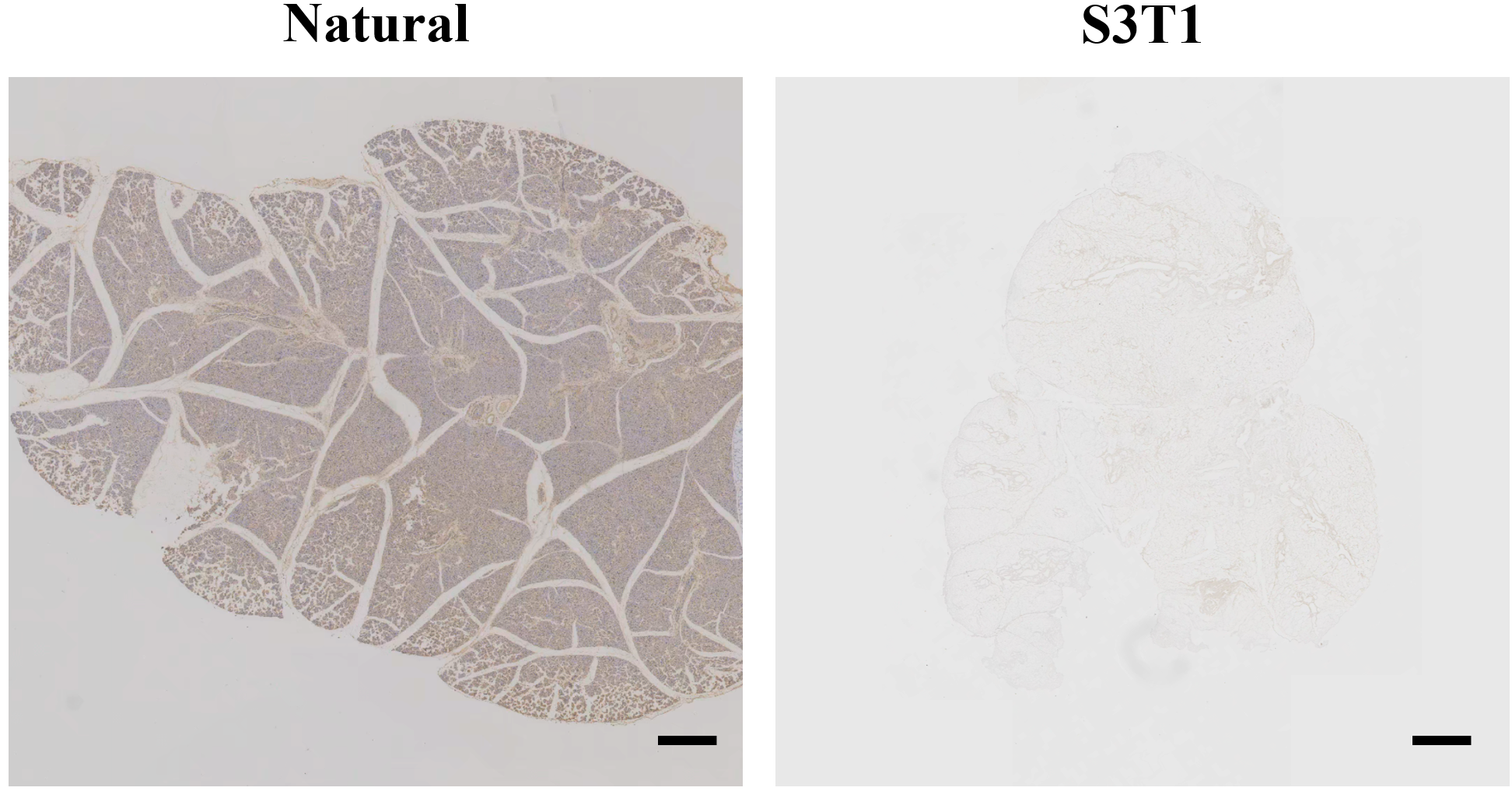


**Supplementary Figure 3. Immunohistochemical staining for α-Gal in native SMG and S3T1 scaffolds.** Native SMG showed strong α-Gal immunoreactivity, whereas staining was markedly reduced in the S3T1 group after decellularization. Scale bars, 500 μm.


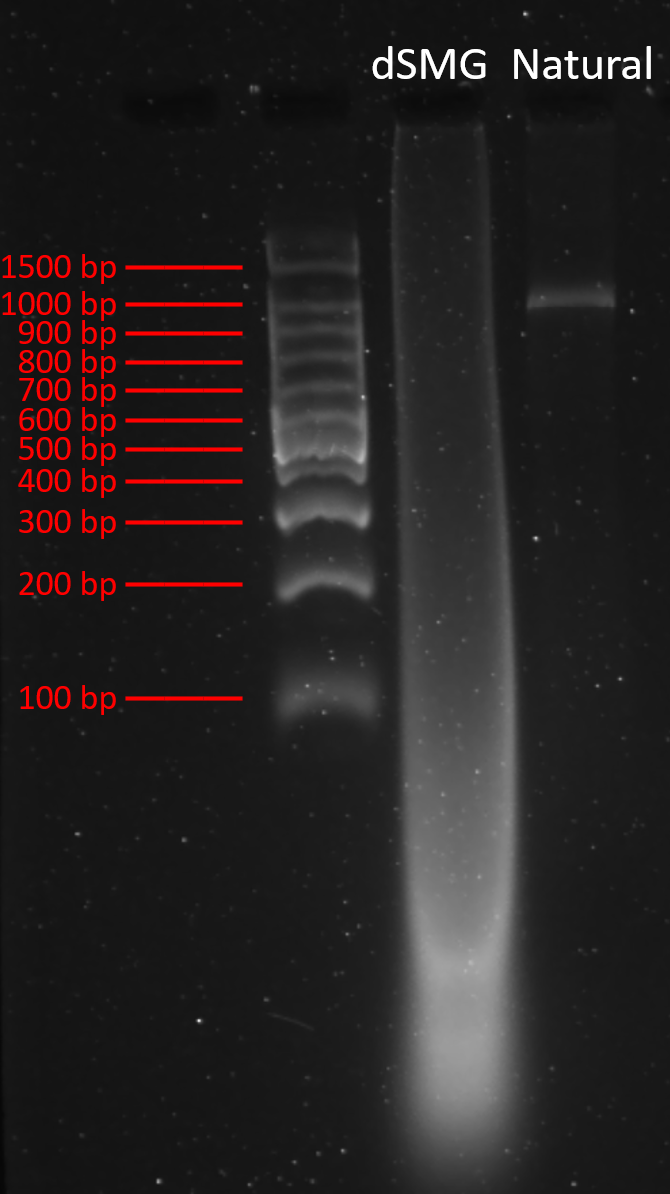


**Supplementary Figure 4. Agarose gel electrophoresis analysis of DNA in native SMG and decellularized SMG, dSMG.** DNA extracted from native submandibular gland tissue, Natural, and decellularized submandibular gland scaffold, dSMG, was separated by agarose gel electrophoresis with a DNA ladder as the size marker.


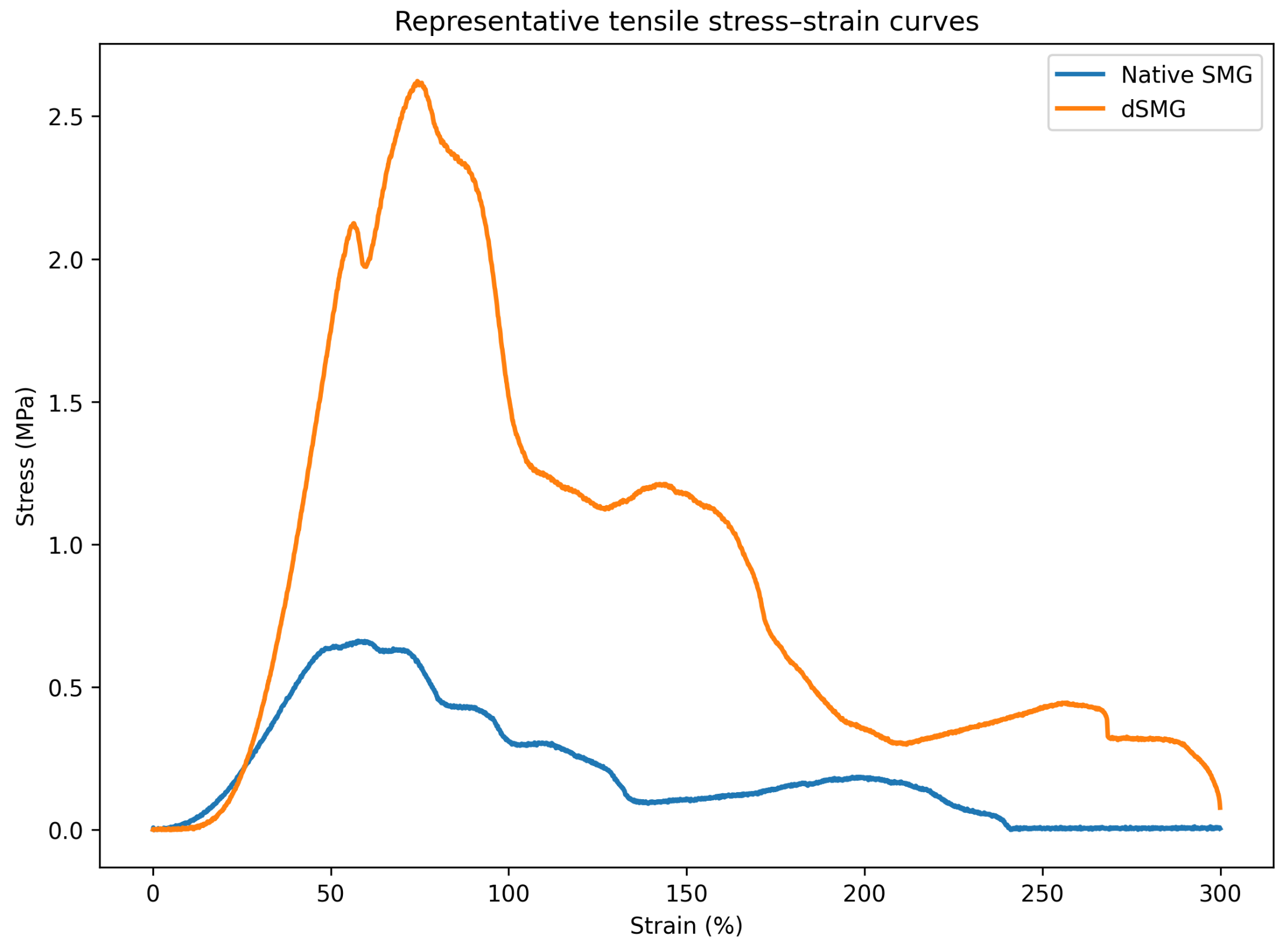


**Supplementary Figure 5. Representative tensile stress–strain curves of native SMG and decellularized SMG scaffold, dSMG.** Uniaxial tensile testing showed that dSMG retained measurable mechanical integrity after decellularization, while exhibiting altered tensile behavior compared with native SMG.


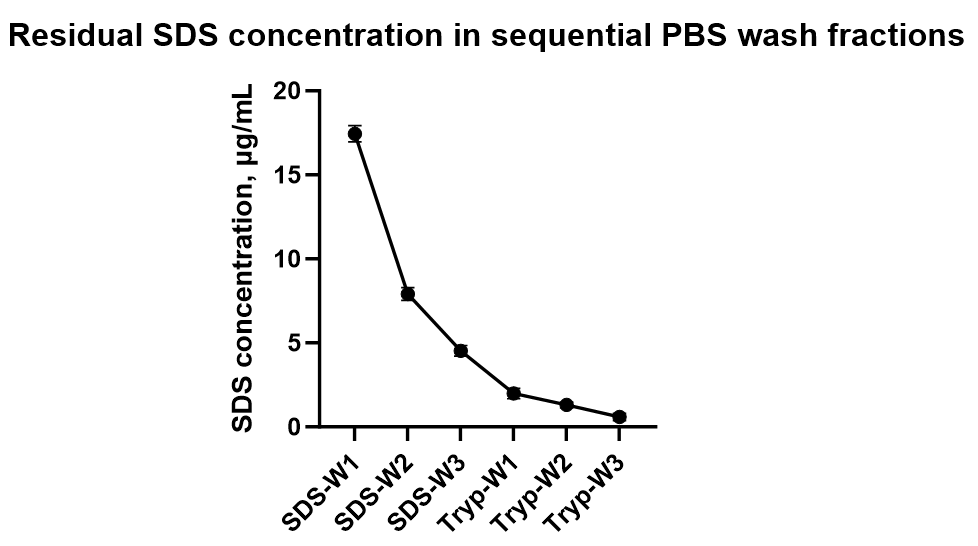


**Supplementary Figure 6. Residual SDS concentration in sequential PBS wash fractions during decellularization.** PBS wash solutions were collected every 10 min during the 30 min wash after 1% SDS treatment, SDS-W1 to SDS-W3, and during the 30 min wash after 0.25% trypsin-EDTA treatment, Tryp-W1 to Tryp-W3. SDS concentration in each fraction was measured using an SDS assay kit. Residual SDS concentration decreased progressively over the washing process. Data are presented as mean ± SD, n = 3.


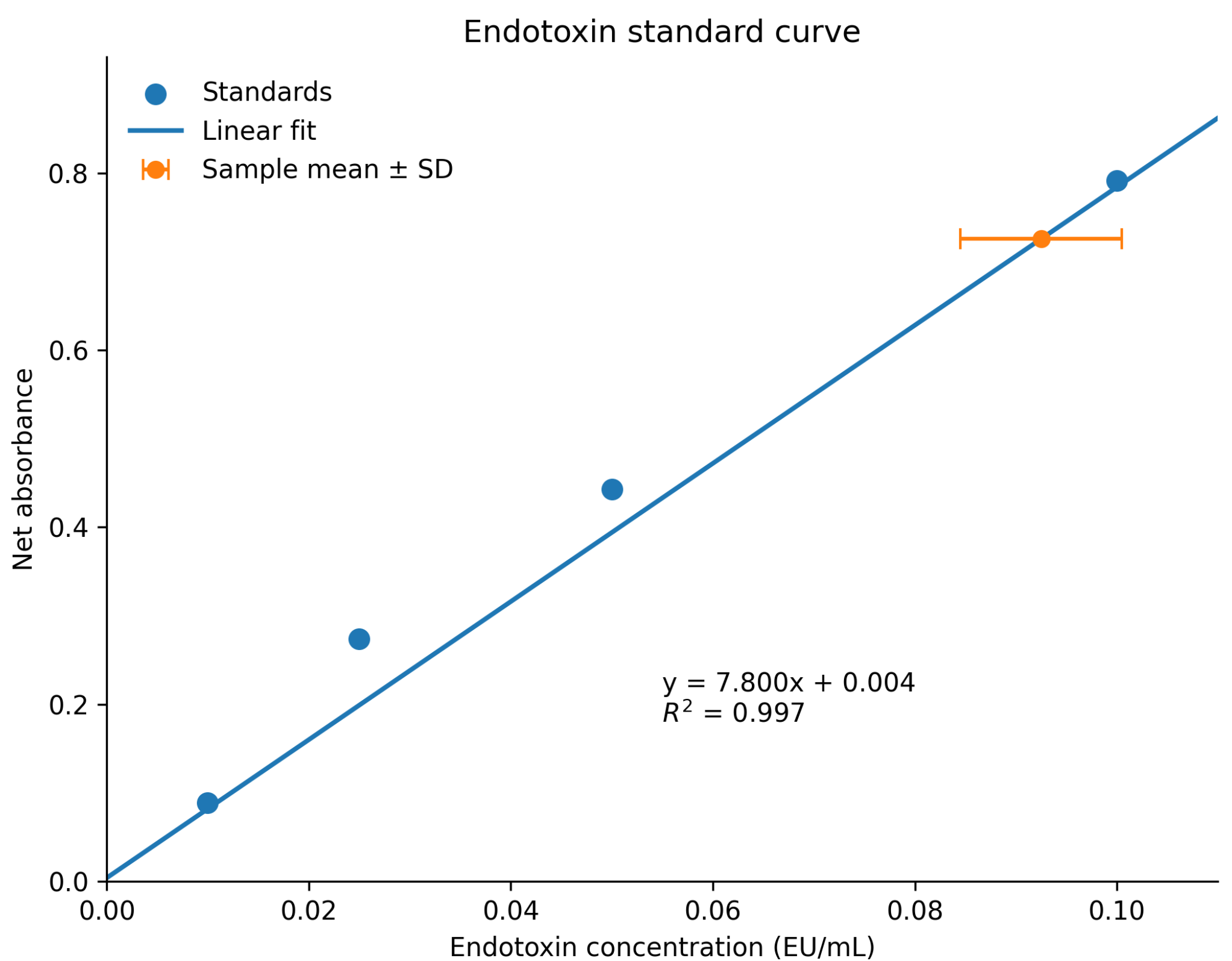


**Supplementary Figure 7. Endotoxin standard curve for quantification of scaffold extract endotoxin levels.** Net absorbance was calculated by subtracting the blank OD value from the measured OD. A linear standard curve was generated using endotoxin standards of 0.01–0.1 EU/mL, with the regression equation **y = 7.800x + 0.004** and **R² = 0.997**. The sample mean ± SD is shown on the curve, n = 3.


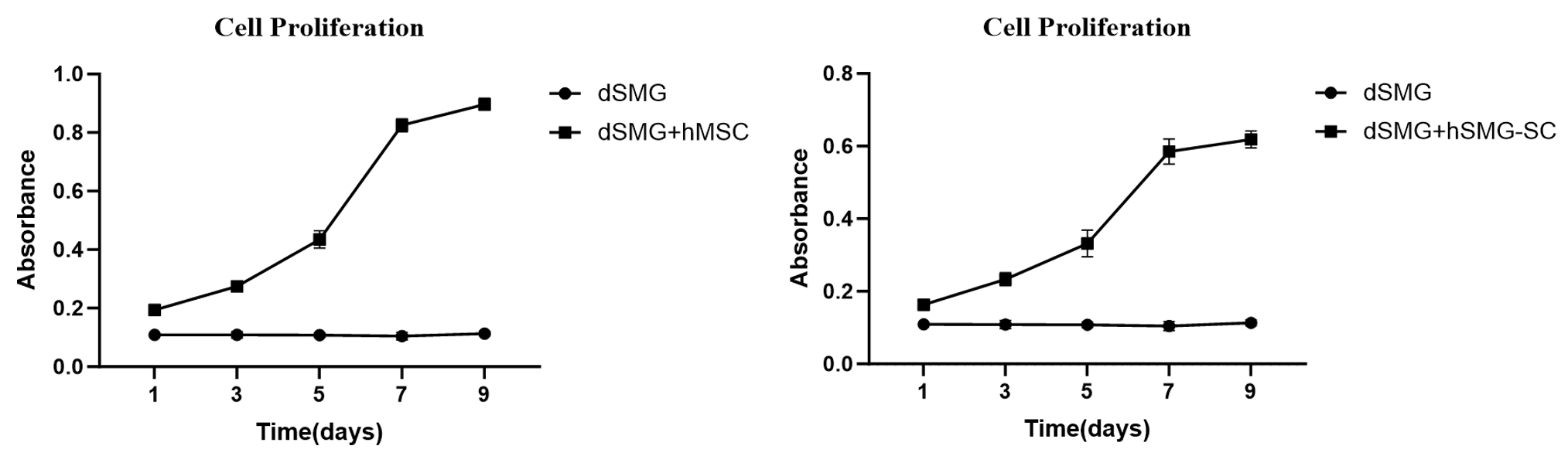


**Supplementary Figure 8. CCK-8 assay of cell proliferation on the surface of washed dSMG scaffolds.** Metabolic activity was measured from day 1 to day 9 in dSMG scaffolds surface-seeded with hMSCs, left, or hSMG-SCs, right, with acellular dSMG serving as the control. Data are presented as mean ± SD, n = 3.


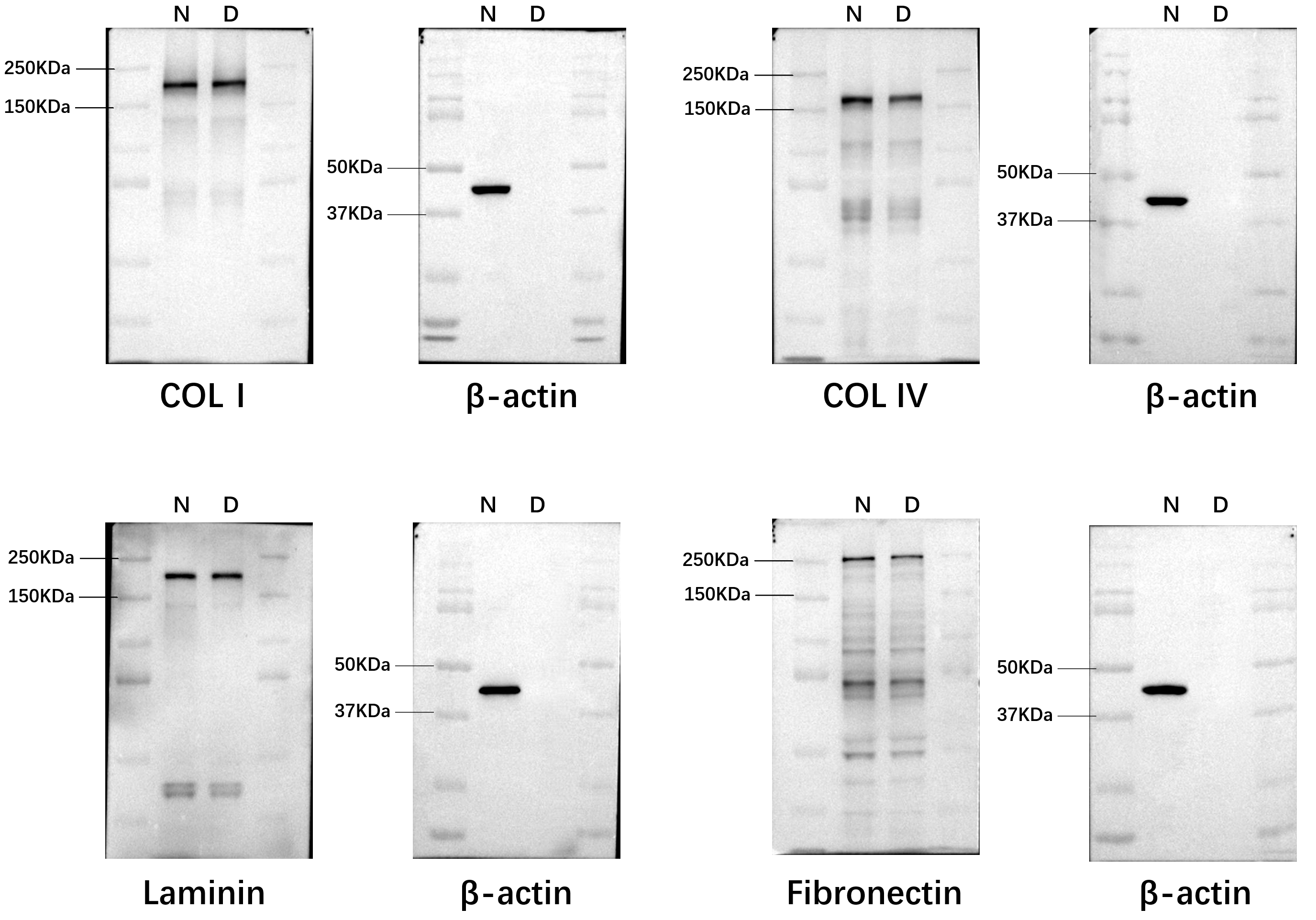


**Supplementary Figure 9. Full-length western blot images of ECM proteins in native and decellularized SMG.** Uncropped western blots of collagen I (COL I), collagen IV (COL IV), laminin, and fibronectin in native submandibular gland (N) and decellularized submandibular gland (D). β-actin was used as the loading control. Corresponding cropped blots are shown in Figure 2C.


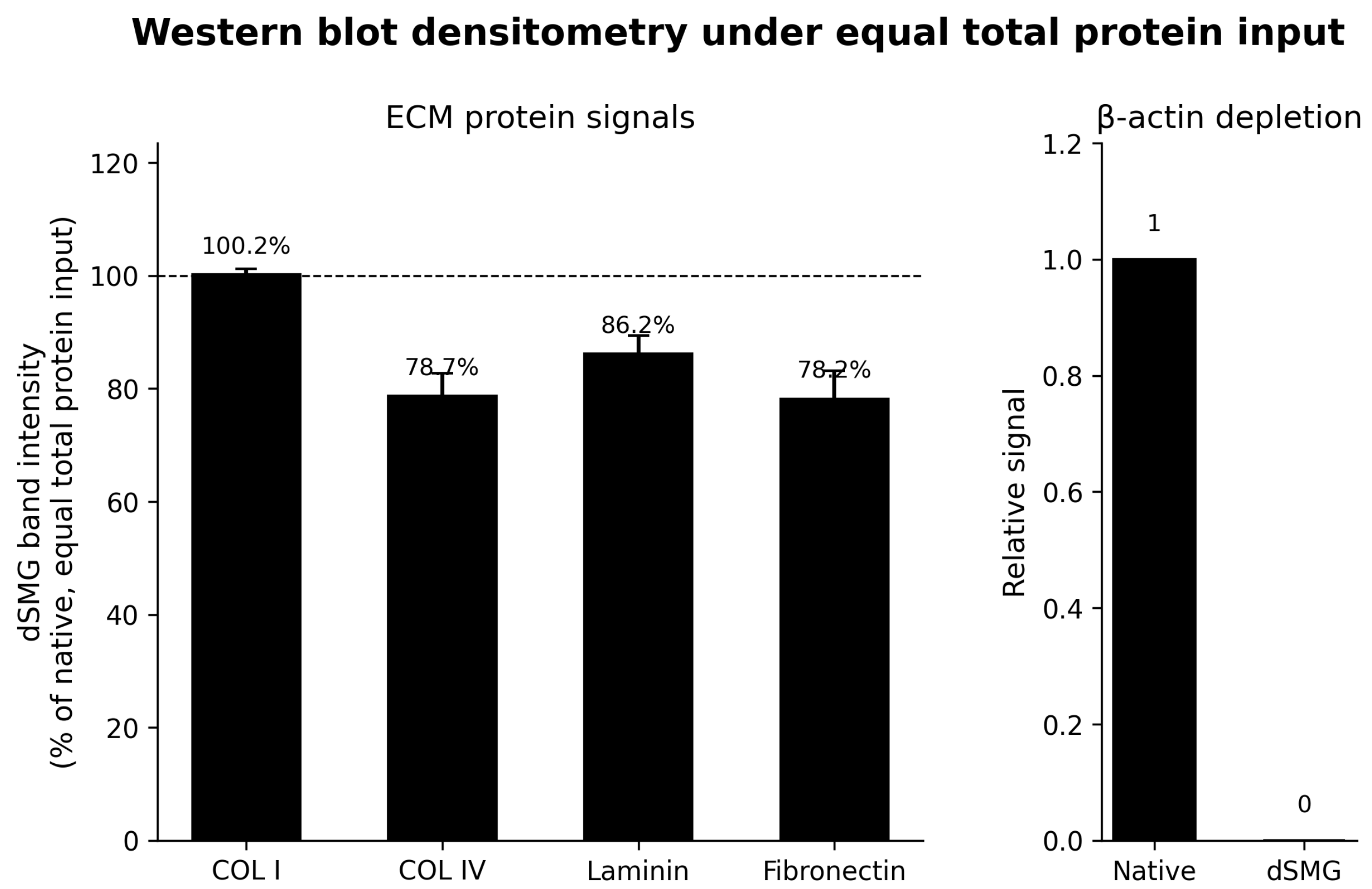


**Supplementary Figure 10.** Western blot densitometry under equal total protein input, 30 μL per lane. Left, ECM protein signals in dSMG relative to native tissue under equal total protein loading, shown as band intensity percentages for COL I, COL IV, Laminin, and Fibronectin. Right, β-actin signal is shown separately as a marker of cellular protein depletion after decellularization rather than as a normalization control. Data are presented as mean ± SD, n = 3.
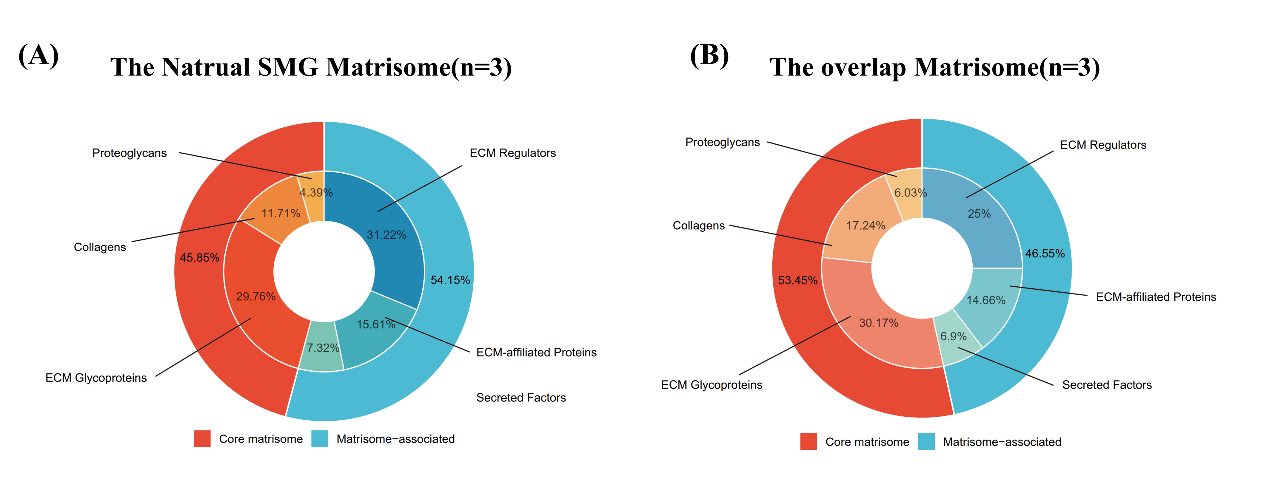


**Supplementary Figure 11. Comparative matrisome composition of natural SMGs and overlap.** Classification of the natural SMG and overlap matrisome into six subcategories according to the Matrisome database.


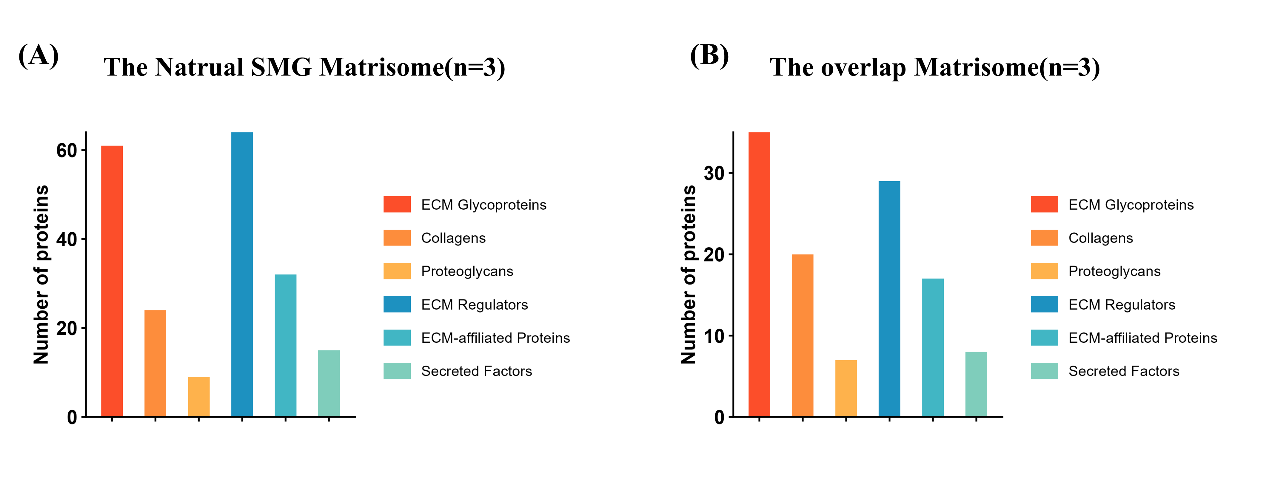


**Supplementary Figure 12. Quantitative comparison of matrisome protein subcategories. Quantitative distribution of ECM protein categories. Proteomic analysis was performed using three biological replicates per group, and data are presented as mean ± SD (n = 3).**


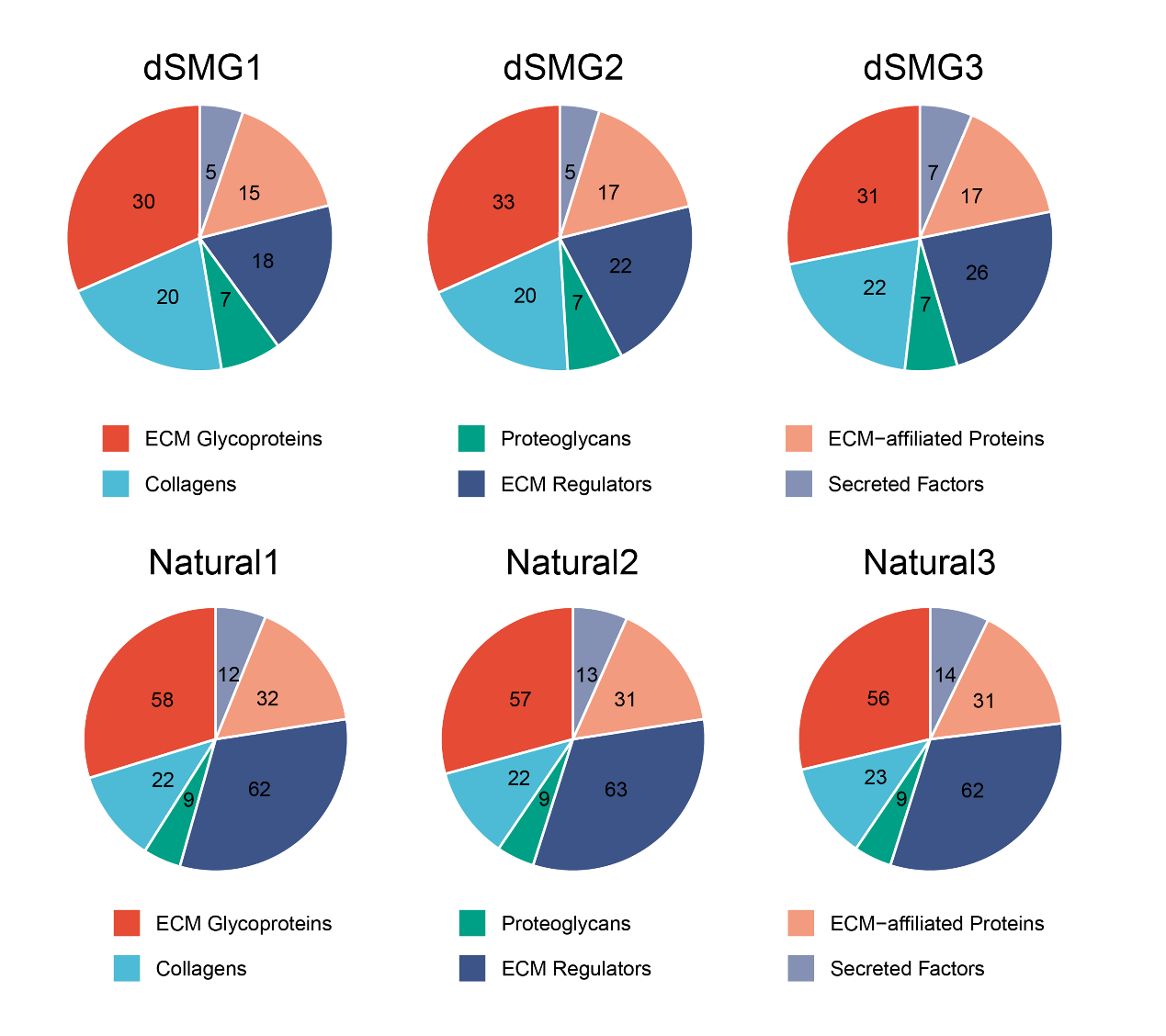


**Supplementary Figure 13. Characterizations of batch variations using the natural SMG and dSMG derived from three different donors. The compositions of matrisome proteins in the six sub-categories are shown for three biological replicates per group (n = 3).**


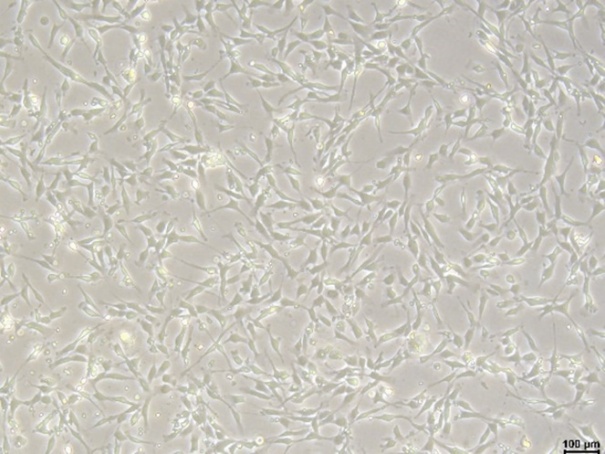


**Supplementary Figure 14.** Representative phase-contrast microscopy image showing passage 3 human mesenchymal stem cells (hMSCs). Scale bar: 100 μm.


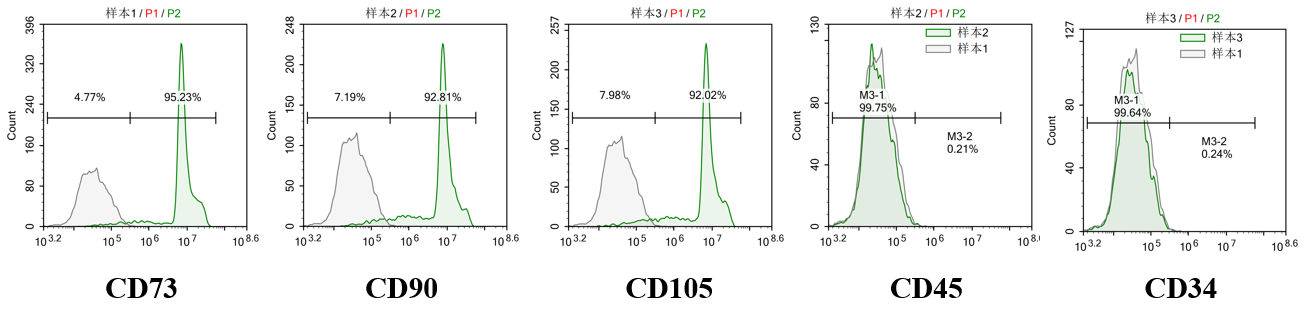


**Supplementary Figure 15.** Flow cytometric analysis of hMSCs showing high expression of mesenchymal markers CD73, CD90, and CD105, and low expression of hematopoietic markers CD45 and CD34.


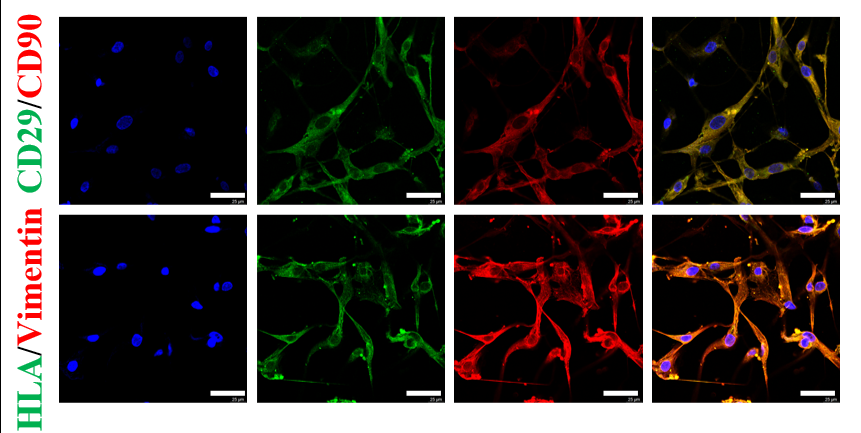


**Supplementary Figure 16.** Immunofluorescence staining confirming the mesenchymal phenotype and human origin of hMSCs, showing positive expression of CD29 (green), CD90 (red), Vimentin (green), and HLA (red). Nuclei were counterstained with DAPI (blue). Scale bars: 50 μm.


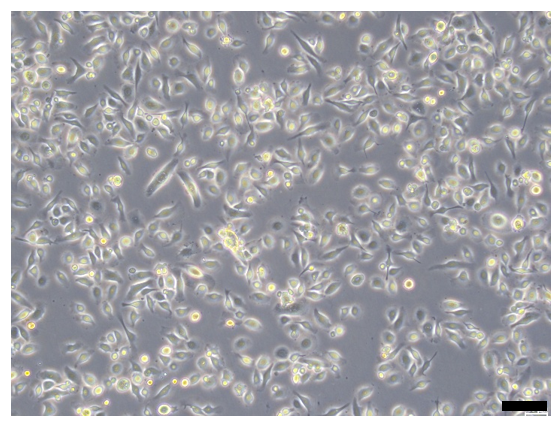


**Supplementary Figure 17.** Representative phase-contrast micrograph of passage-3 hSMG-SCs showing typical epithelial-like morphology. Scale bar, 200 μm.


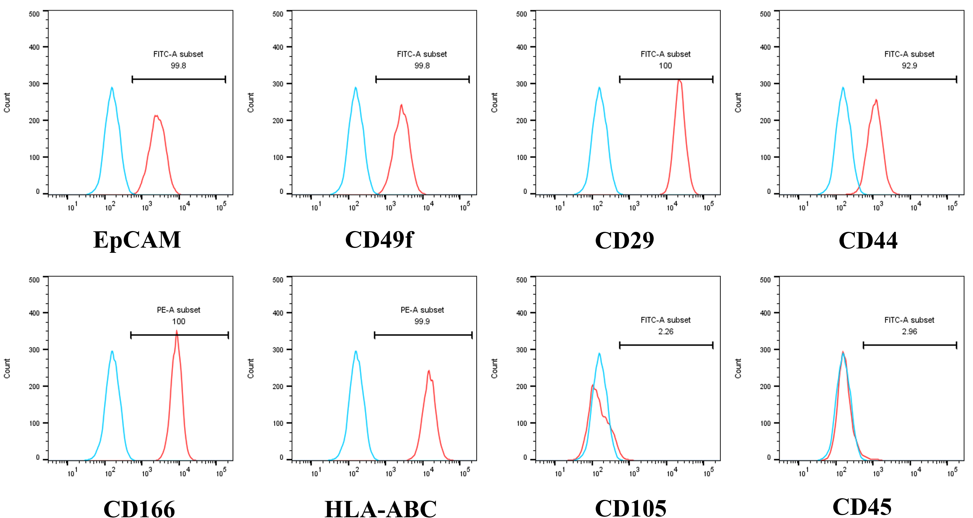


**Supplementary Figure 18.** Flow cytometry analysis of hSMG-SC surface marker expression showing positivity for epithelial-associated markers (EpCAM, CD49f, CD29, CD44, CD166, and HLA-ABC) and lack of expression of mesenchymal and hematopoietic markers CD105 and CD45.


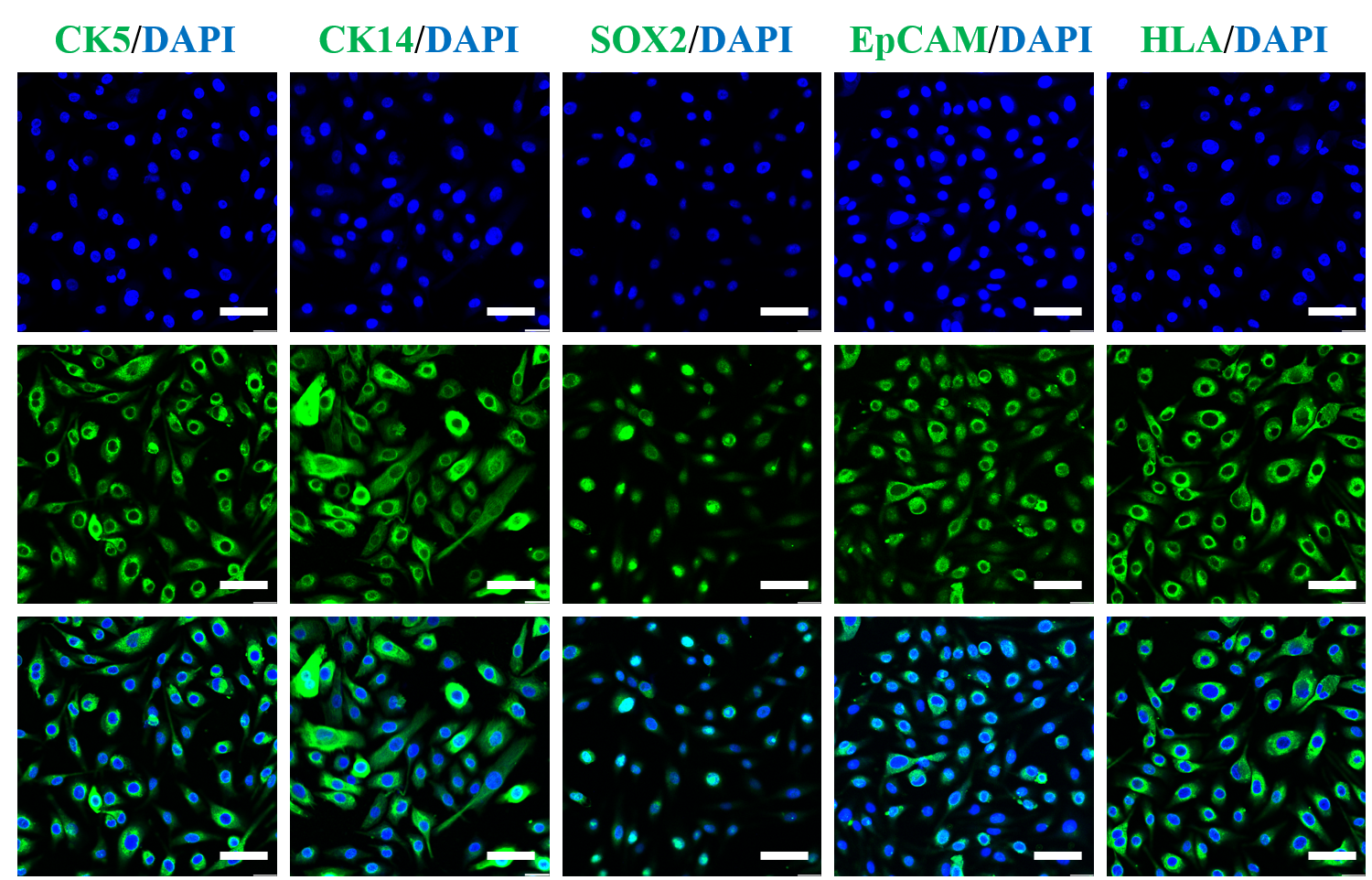


**Supplementary Figure 19.** Immunofluorescence staining of hSMG-SCs showing positive expression of CK5, CK14, SOX2, EpCAM and HLA, with nuclear counterstaining by DAPI. Scale bars, 50 μm.


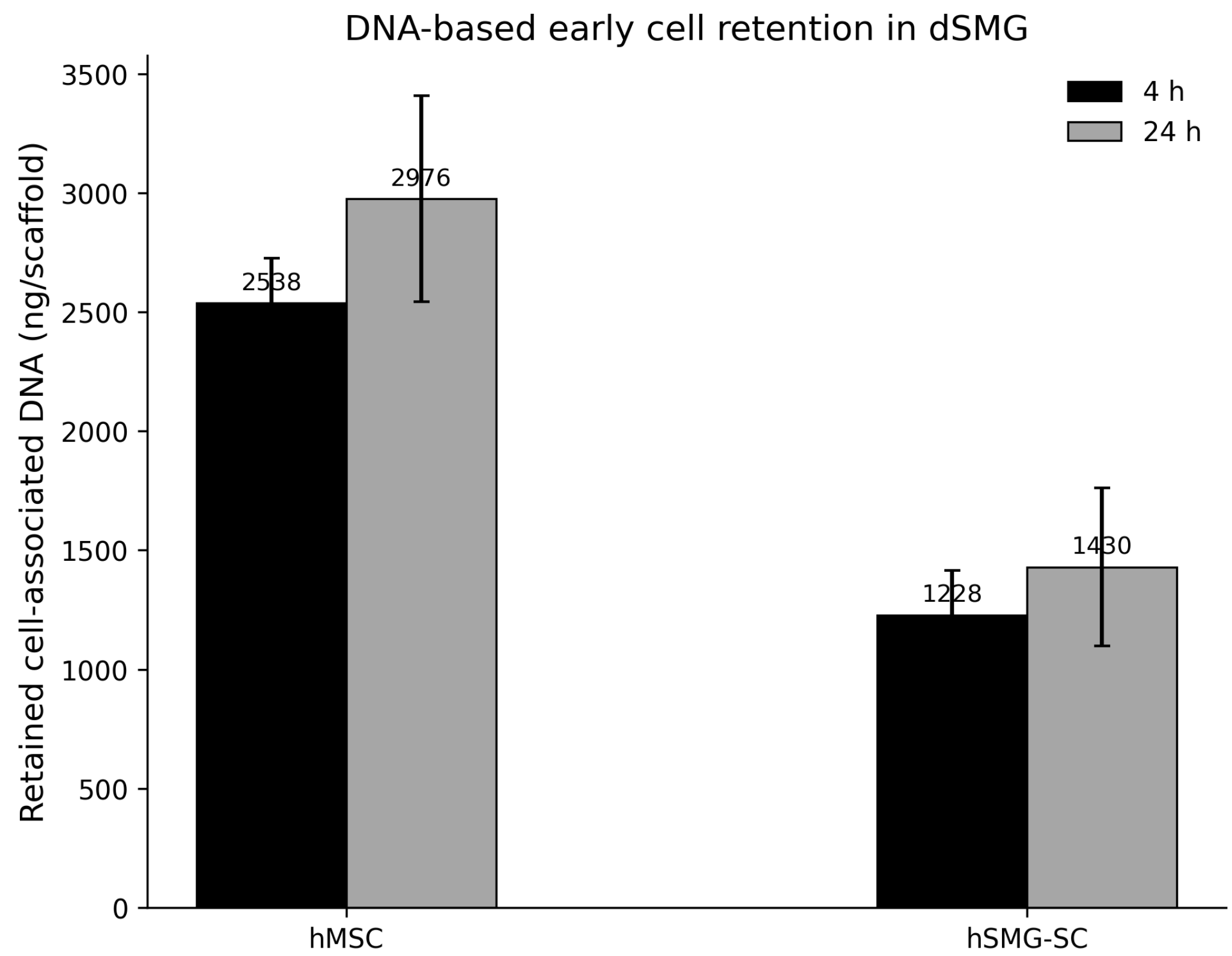


**Supplementary Figure 20. DNA-based evaluation of early cell retention in dSMG scaffolds after intraductal delivery of hMSCs or hSMG-SCs.** Retained cell-associated DNA was quantified at 4 h and 24 h after seeding by subtracting the background DNA content of acellular dSMG scaffolds from the total DNA measured in recellularized scaffolds. Data are presented as mean ± SD, n = 3.


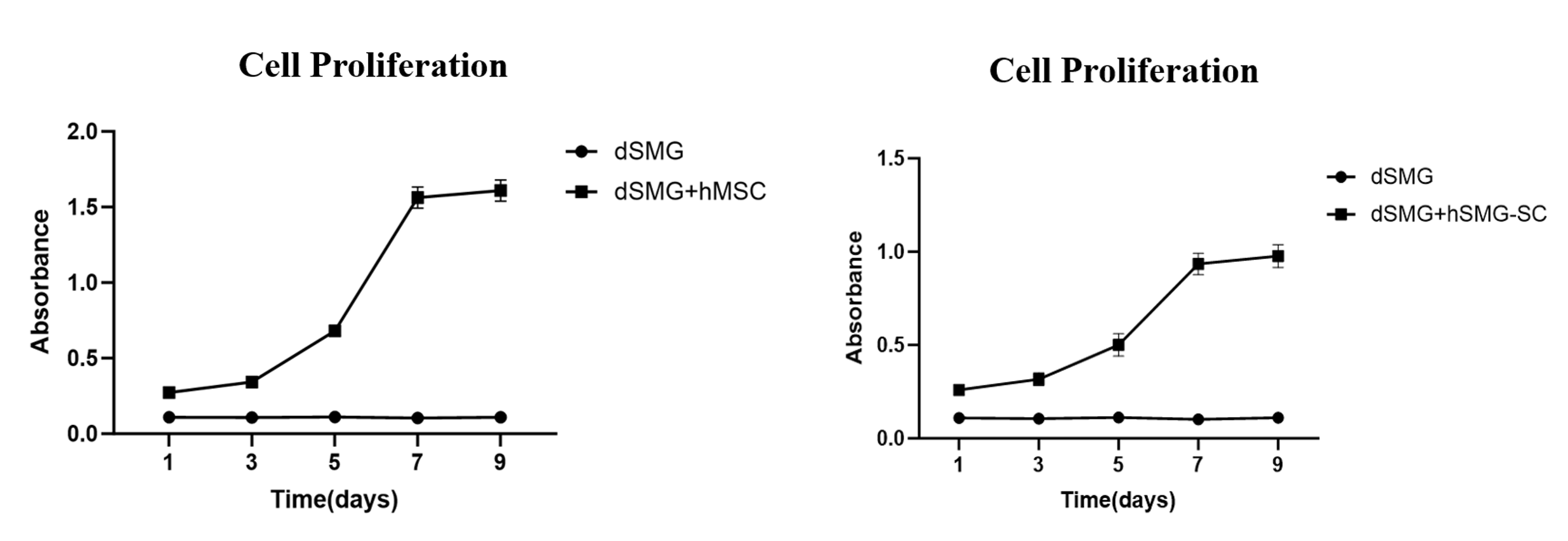


**Supplementary Figure 21. Cell proliferation assays (CCK-8) of dSMG scaffolds recellularized with hMSCs or hSMG-SCs.** Metabolic activity was measured from day 1 to day 9 in dSMG seeded with hMSCs (left) or hSMG-SCs (right), with acellular dSMG serving as the control. Data are presented as mean ± SD (n = 3).


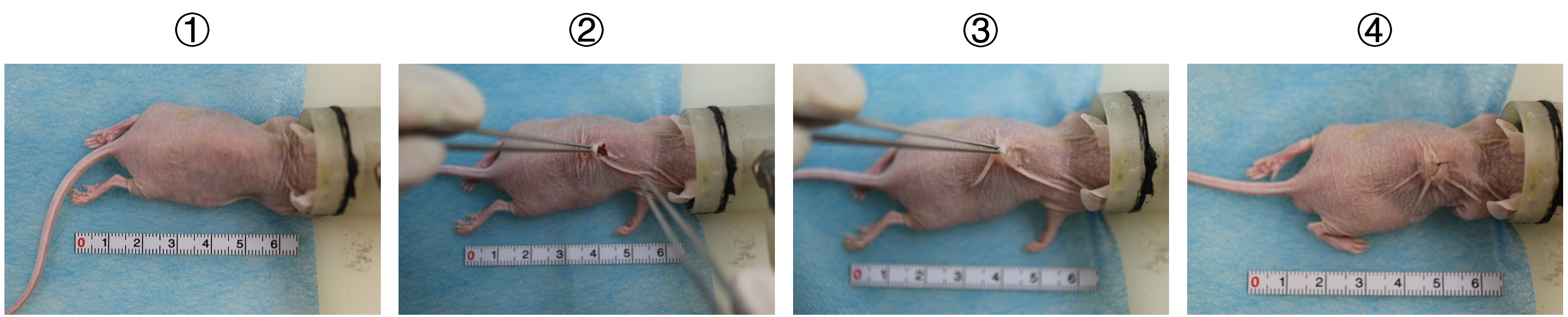


**Supplementary Figure 22. Surgical procedure for subcutaneous implantation of dSMG scaffolds in nude mice.** ① The nude mouse was placed in the prone position under anesthesia and the surgical field was prepared. ② A small dorsal incision was made and a subcutaneous pocket was bluntly dissected. ③ The dSMG scaffold was inserted into the subcutaneous space using forceps. ④ The incision was closed after implantation, completing the procedure.


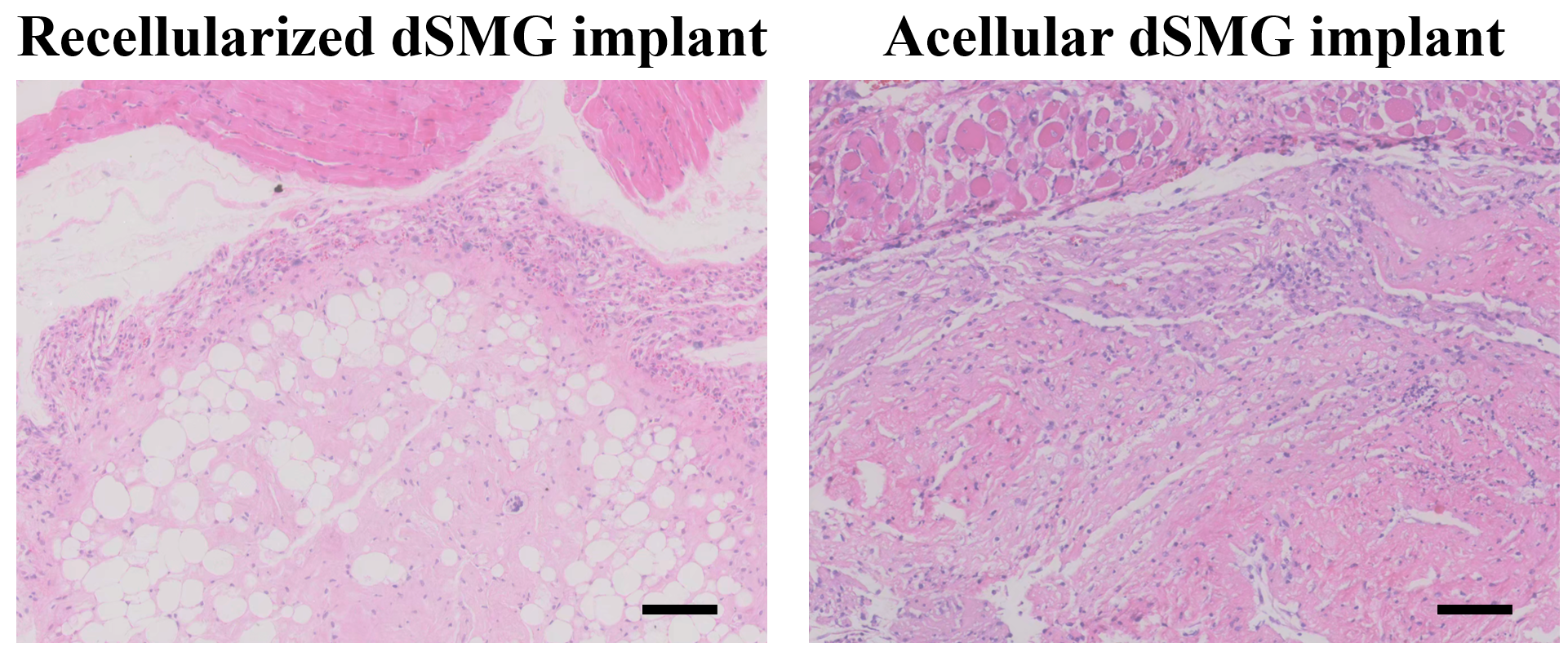


**Supplementary Figure 23.** Representative H&E images showing inflammatory reactions at the interface between the implanted scaffold and adjacent skin tissue in the recellularized dSMG implant and acellular dSMG implant groups. Both groups showed mild-to-moderate inflammatory cell infiltration at the host–implant interface, without evidence of severe inflammatory reaction. Scale bars, 100μm.


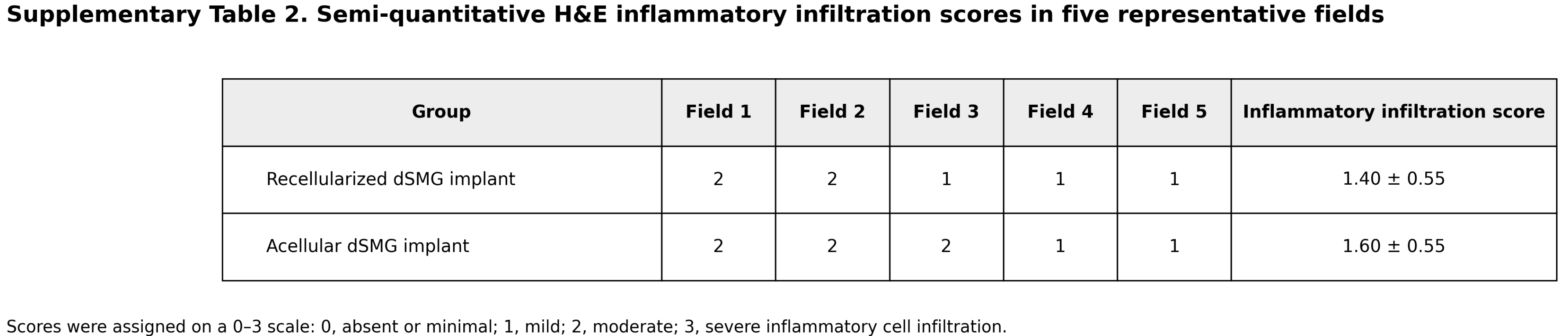

Supplement: Supplementary file 1 [file Supplementaryfile1.docx]
